# A bispecific antibody designed to act as a NRP2/PLXNA1 agonist mimics anticancer activity of SEMA3F

**DOI:** 10.1016/j.jbc.2025.111056

**Published:** 2025-12-13

**Authors:** Honglei Tian, Chun Po Fung, Luke Burman, Yeeting E. Chong, Changdong Liu, Yanyan Geng, Lam Yang, Man Wai Chow, Yingyi Zhang, Kwok Wa Hugo Ho, Guang Zhu, Zhenguo Wu, Xiang-Lei Yang, Zhiwen Xu, Leslie A. Nangle

**Affiliations:** 1 IAS HKUST-Scripps R&D Laboratory, Institute for Advanced Study, Hong Kong University of Science and Technology, Kowloon, Hong Kong, China; 2 Division of Life Science, The Hong Kong University of Science and Technology, Hong Kong, China; 3 Pangu Biopharma, Hong Kong, China; 4 aTyr Pharma, San Diego, California, USA; 5 Biological Cryo-EM Center, The Hong Kong University of Science and Technology, Hong Kong, China; 6 Department of Integrative Structural and Computational Biology, The Scripps Research Institute, La Jolla, California, USA

**Keywords:** semaphorin 3F, bispecific antibody, PLXNA1, NRP2, anticancer, tumor suppression, receptor dimerization, phospho-AKT, oncogene expression, cell proliferation, cryo-EM, single B-cell platform, antibody discovery, U-251 MG cells, SEMA3F-mimetic

## Abstract

Neuropilin-2 (NRP2) is a pleiotropic receptor with diverse roles across biological systems. Recent work detailed its role as an immunomodulatory receptor target that is currently being explored in clinical development for interstitial lung diseases, establishing it as a viable therapeutic target. To mediate its diverse effects, NRP2 interacts with endogenous ligands, including semaphorins (SEMAs) and vascular endothelial growth factors, signaling *via* ligand-induced heterodimerization with various receptor families. One of these ligands, SEMA3F exhibits well-documented tumor-suppressive activities mediated through NRP2 and plexinA1 (PLXNA1). Despite its observed benefits, SEMA3F is not therapeutically viable due to the multifaceted nature of its functions through non-NRP2–mediated interactions, leading to concerns around potential toxicity. Here, we describe development of bispecific antibodies (bsAbs) that dimerize PLXNA1 and NRP2, selectively mimicking the beneficial aspects of SEMA3F signaling as a basis for a novel anticancer therapy. Using a single B cell–based mAb discovery platform, anti-PLXNA1 mAbs with diverse lineages were generated and combined with anti-NRP2 mAbs to produce over 200 PLXNA1-NRP2 bsAbs. Antibodies were screened in cell-based assays (receptor dimerization, phospho-AKT, oncogene expression, and cell proliferation), yielding one bsAb capable of mimicking NRP2-mediated SEMA3F activities in all assays. Structural studies revealed that this bsAb binds to PLXNA1/NRP2 at sites distinct from the SEMA3F-binding site, but in a manner that allows proper spacing for receptor complex formation and flexibility of conformational changes for signaling. This study demonstrates the potential of these receptors as targets for agonistic bsAbs development and provides the groundwork for further exploration in tumor models.

Semaphorins (SEMAs) are a group of regulatory proteins that are found in viruses, invertebrates and vertebrates in both secreted and membrane-bound forms. All SEMAs are comprised of a sema domain, which is characterized by a seven-blade beta propeller fold structure that serves as a receptor recognition and binding module (1). In vertebrates, five classes of SEMAs have been identified based on their sequence similarity and structural characteristics, and are designated as class 3 to 7 SEMAs (2). Among these vertebrate SEMA classes, class 3 SEMAs are secreted proteins, which are comprised of a SEMA domain, a plexin-semaphorin-integrin (PSI) domain, and an immunoglobulin-like (Ig-like) domain (2, 3). SEMAs are expressed in a wide range of tissues and are primarily involved in regulating cell attachment and motility in physiological and pathological processes such as axon guidance, immune cell regulation, vascular growth, and tumor progression (4). Plexin proteins are the main receptors for SEMAs (5, 6, 7). A total of 9 plexin proteins have been identified in vertebrates and are classified into four subfamilies: plexinA to plexinD (6). Plexins (PLXNs) contain SEMA and PSI domains as well as additional Ig-like, PLXNs and transcription factors (IPT) domains (4). PLXNs generally function as single pass transmembrane receptors with a GTPase-activating protein domain in the intracellular portion which is thought to dimerize with Rap. Neuropilin (NRP) is another receptor family for SEMA, which is specific to class 3 SEMAs (8, 9). Two family members of NRP have been identified, NRP1 and NRP2 (10, 11), which are composed of two CUB (a1/a2) domains, two factor V/VIII homology (b1/b2) domains, one MAM (c) domain, one transmembrane domain, and a short intracellular sequence (43–44 amino acid long) (12). NRPs are pleiotropic receptors involved in multiple cellular signaling for diverse biological functions (13). Notably, NRP2 is currently being explored as an immunomodulatory receptor target in clinical development for interstitial lung diseases (14).

SEMA3F, a member of class 3 SEMA family proteins, has emerged as an anticancer regulator with significant implications in cancer biology. The *SEMA3F* locus was first identified within an allele with high frequency of deletion in human lung tumors (15, 16, 17). Subsequently, the *SEMA3F* gene was found to be a tumor suppressor. Transfection of this allele or SEMA3F complementary DNA (cDNA) into cancer cells inhibits proliferation of various types of cancer cells and mouse xenograft tumor, including lung cancer (18, 19, 20), melanoma (21), fibrosarcoma (22, 23), breast cancer (24), glioma (25), osteosarcoma (26), esophageal squamous cell carcinoma (27), and head and neck squamous carcinoma (28). SEMA3F also plays a pivotal role in migration and metastasis of cancer cells. Knockdown of SEMA3F leads to an increased migration and metastasis of osteosarcoma SaOS and MG-63 cells (26) and spleen-xenografted HCT116 colon cancer cells (29). Conversely, overexpression of SEMA3F has been shown to inhibit migration and invasion of esophageal squamous cell carcinoma Eca109 and TE1 cells (27). In addition, an elevation of SEMA3F has been associated with a decrease in lymph node metastasis and lymphangiogenesis of head and neck squamous cell carcinoma (28). Furthermore, higher expression of SEMA3F is correlated with better outcomes for various types of cancer patients, including osteosarcoma (26), esophageal cancer (27), breast cancer (30), head and neck squamous carcinoma (28), colorectal cancer (29, 31, 32), and prostate cancer (33). In terms of the molecular mechanism of its anticancer activity, SEMA3F’s signaling and biological response are primarily mediated through its interaction with NRP2 and PLXNs (28). Binding of SEMA3F to NRP2 and PLXNA1 promotes collapse of COS-7 and lymphatic endothelial cells (28). SEMA3F also induces formation of NRP2–PLXNA1 complex, which recruits ABL2 and p190 to inactivate RhoA, inhibiting cell migration and contractility of U87 MG glioma cells (34). In a mouse xenograft model, SEMA3F inhibits AKT/mTOR signaling pathway to reduce human glioma cell growth (25). SEMA3F also downregulates ASCL2–CXCR4 axis through the PI3K–AKT pathway (29) and targets Wnt/β-catenin pathway through GTP-Rac1 (32) to suppress the invasion and metastasis of colorectal cancer cells.

Despite their documented anticancer properties, class 3 SEMAs including SEMA3F are not ideal for drug development due to limitations in selectivity and stability. First, they lack binding selectivity and interact with multiple receptor targets including PLXNs A-D, NRP1/2, receptor tyrosine kinases and integrins, which can lead to broad signaling outcomes with potential adverse effects (35). For example, SEMA3A promotes clonogenic growth of glioblastoma by the activation of NRP1 pathway (1) and contributes to ischemia-induced brain damage through binding to NRP2/VEGFR1 receptor complex (36). Although SEMA3F inhibits growth, metastasis and invasion of breast, lung and colorectal cancers, it also promotes metastasis and invasion of hepatocellular carcinoma through unknown targets that distinctly activate the focal adhesion pathway (37). Second, class-3 SEMAs suffer from poor stability and pharmacokinetic properties. The proteins are susceptible to cleavage by the proprotein convertase furin that is ubiquitously expressed by mammalian cells and present in circulation. The cleavage of class-3 SEMAs at major sites usually leads to inactivation (38, 39). For SEMA3F in particular, the short half-life combined with its large size (∼90 kDa) and complex structure, limits its tissue penetration and bioavailability diminishing the therapeutic window (40). Alternatively, a bispecific antibody (bsAb) that selectively binds and dimerizes PLXNA1 and NRP2 has the potential to mimic the anticancer activities of SEMA3F, reducing potential side effects by exclusively targeting these receptors. In addition, the modular design of bsAbs allows for good stability and favorable pharmacokinetic properties (41) creating the basis for the potential development of a viable therapeutic candidate.

In this study, we aimed to screen for and identify bsAbs that dimerize NRP2 and PLXNA1 to mimic SEMA3F in cellular signaling and activities as anticancer therapeutic candidates. To achieve this goal, we first identified cancer cell types and assays that demonstrated SEMA3F-mediated signaling along with its anticancer effects. Next, we identified a panel of anti-PLXNA1 (aPLXNA1) mAbs with diverse lineages using a single B cell–based mAb discovery platform, in which antibody genes are directly cloned from antibody-secreting cells to allow the discovery of highly diversified as well as rare mAbs. These aPLXNA1 mAbs were combined with 4 anti-NRP2 (aNRP2) mAbs, which were previously discovered and characterized with distinct binding domains on NRP2 (42), to generate over 200 PLXNA1-NRP2 bsAbs using a knobs-into-holes strategy (43, 44). For downstream screening of bsAbs with SEMA3F-mimetic anticancer activities, we employed four cell-based assays: receptor dimerization, phospho-AKT, oncogene expression, and cell proliferation to assess the functional effects of each bsAb. One bsAb replicated SEMA3F activity in all four cell-based assays. To explore its mechanism, we conducted cryo-EM structural studies to map epitope sites and elucidate the molecular basis of bsAb-induced PLXNA1-NRP2 dimerization underlying its SEMA3F-mimetic effects.

## Results

### SEMA3F inhibits glioblastoma U-251 MG cells proliferation in an NRP2-dependent manner

**Figure 1:**
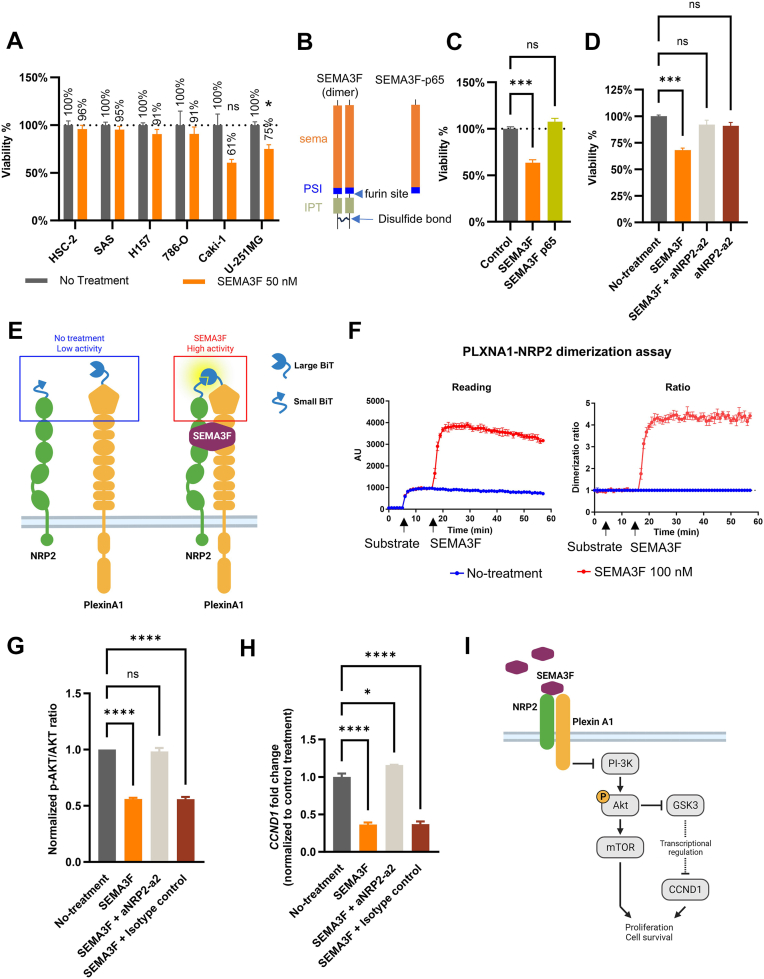
**SEMA3F dimerizes surface NRP2 and PLXNA1, inhibits U-251 MG cell proliferation, and reduces p-AKT level and *CCND1* gene expression**. *A*, effects of SEMA3F on proliferation of various cancer cell lines. Cells were treated with full-length SEMA3F for 3 days, and cell viability was measured using CellTiter-Glo, normalized to untreated cells. *B*, schematic illustrates the full-length SEMA3F dimer, the furin processing site, and the resulting furin-processed SEMA3F (SEMA3F-p65). *C*, SEMA3F-p65 does not inhibit U-251 MG cell proliferation compared to nonprocessed SEMA3F dimer. *D*, the anti-NRP2 antibody (aNRP2-a2) blocks SEMA3F-mediated inhibition of U-251 MG cell proliferation. *E*, schematic illustrating the receptor dimerization assay. NanoLuc luciferase is split into Large BiT and Small BiT with low activity, and fused to the N termini of NRP2 and PLXNA1. SEMA3F induces NRP2-PLXNA1 dimerization, bringing Large BiT and Small BiT together to enhance luciferase activity through complementation. *F*, SEMA3F treatment induces NRP2-PLXNA1 dimerization. *Left panel*: Expi293F cells coexpressing Large_BiT-NRP2 and Small_BiT-PLXNA1 were treated with luciferase substrate and SEMA3F as indicated, and luminescence was recorded over time. *Right panel*: dimerization ratio changes following SEMA3F treatment. *G*, SEMA3F reduces p-AKT levels in U-251 MG cells in an NRP2-dependent manner. U-251 MG cells were treated as indicated for 30 min, and the p-AKT/AKT ratio was measured. *H*, SEM3F downregulates *CCND1* expression in an NRP2-dependent manner. U-251 MG cells treated for 18 h. The expression of *CCND1* was quantified using qPCR and normalized to untreated control cells. *I*, schematic representation of SEMA3F’s anti-proliferative mechanism in U-251 MG cells. NRP, neuropilin; aNRP2, anti-NRP2; PLXNA1, plexinA1; SEMA, semaphorin; p-AKT, phosphorylation of AKT; qPCR, quantitative PCR.

SEMA3F has been reported to inhibit proliferation of various types of cancer cells. We tested the inhibitory effect of SEMA3F on proliferation of 6 cancer cell lines available in house, including 3 oral cancer cell lines (HSC-2, SAS, and H157), 2 renal cancer cell lines (786-O, and Caki-1), and 1 glioblastoma cell line (U-251 MG) (Table S1). SEMA3F significantly inhibited the proliferation of U-251 MG cells (Fig. 1*A*). Although SEMA3F also suppressed Caki-1 cell proliferation in trend, it did not reach statistical significance. Based on the Human Protein Atlas (https://www.proteinatlas.org/), the U-251 MG cell line has high expressions of both PLXNA1 and NRP2, whereas the other cell lines have less (Table S2). This supports SEMA3F exerts anticancer activities through PLXNA1/NRP2, and provides justification for U-251 MG’s application in screening for PLXNA1-NRP2 bsAbs that mimic SEMA3F. Notably, SEMA3F was cleaved by furin to generate a shorter and monomeric form, SEMA3F-p65 (45, 46) (Fig. 1*B*). In the cell proliferation assay, SEMA3F-p65 lacked the inhibitory effect on U-251 MG cells as expected and was employed as a negative control in this study (Fig. 1*C*). Next, we utilized a previously developed aNRP2 antibody, aNRP2-a2 that targets the a2 domain of NRP2 and blocked the binding of SEMA3F to NRP2 (42), to explore if the cellular activity of SEMA3F was mediated by NRP2. This antibody blocked the inhibitory effect of SEMA3F on U-251 MG cell proliferation (Fig. 1*D*), suggesting that NRP2 is a major receptor that mediates the anticancer activity of SEMA3F on U-251 MG.

### SEMA3F dimerizes NRP2 and PLXNA1 on the cell surface

Structural and biochemical studies revealed that SEMAs induce heterodimerization of PLXNs and NRPs (34, 47, 48). To investigate whether SEMA3F dimerizes cell surface PLXNA1 and NRP2, a receptor dimerization assay based on split NanoBiT luciferase was set up (Fig. 1*E*). PLXNA1 and NRP2 were tagged with each half of the split luciferase and overexpressed in Expi293F cells. In terms of luciferase orientation, both extracellular (N-terminal) and intracellular (C-terminal) tagging of the luciferase were explored (Fig. S1, *A*–*C*). The N-terminal tagging was found to result in a more profound luminescence increase and fold changes than the C-terminal tagging (Fig. S1, *B* and *D*). As shown in Figure 1*F*, SEMA3F induced around 4.3-fold luminescence increase compared to untreated control, demonstrating SEMA3F dimerizes NRP2 and PLXNA1 on the cell surface. As expected, the negative controls with Expi293F expressing only PLXNA1 or NRP2 did not show any response to SEMA3F treatment (Fig. S1*E*). It was also noted that the effect of SEMA3F on PLXNA1-NRP2 dimerization started within several minutes of application and quickly plateaued. This is consistent with previous reports that SEMA3F forms a complex with NRP2 and PLXNA1 within 5 min upon treatment on human glioma cells (34) and induced the reduction in phosphorylation of downstream signaling molecules between 5 and 20 min (25).

### SEMA3F reduces p-AKT level and downregulates oncogene expression in an NRP2-dependent manner in U-251 MG cells

It is known that NRP2 and PLXNA1 dimerization leads to activity inhibition of PI3K and reduces phosphorylation of AKT (p-AKT) (25, 49). As expected, SEMA3F treatment led to a ∼50% reduction of p-AKT level in U-251 MG cells (Fig. 1*G*). This reduction was reversed by the aNRP2-a2 antibody, which prevents interaction between SEMA3F and NRP2, but not by an isotype control antibody indicating the p-AKT reduction was mediated through NRP2 (Fig. 1*G*).

To explore which genes are regulated downstream of p-AKT signaling pathway by SEMA3F treatment, we examined gene expression changes by quantitative PCR (qPCR) arrays. Two panels of genes were screened, including those involved in the PI3K–AKT signaling pathway and its downstream effector mTOR signaling pathway. In total, there were 168 genes included in these two qPCR panels (Tables S3 and S4). Screening results showed that 9 genes had at least 1.5-fold change after SEMA3F treatment (Fig. S1*F* and Tables S3 and S4). Further time-course study demonstrated that the expression of oncogene *CCND1*, which encodes cyclin D1 involved in the cell cycle regulation, was significantly reduced 6 to 24 h post-SEMA3F treatment (Fig. S1*G*). The reduction of *CCND1* level by SEMA3F treatment was reversed by the aNRP2-a2 antibody, indicating this effect was NRP2-dependent (Fig. 1*H*).

The mechanism studies demonstrate that SEMA3F dimerizes surface NRP2 and PLXNA1, inhibiting U-251 MG cell proliferation through the PI3K-AKT and CCND1 pathways (Fig. 1*I*).

### Establishment of mAb discovery platform based on single B cell technology

**Figure 2:**
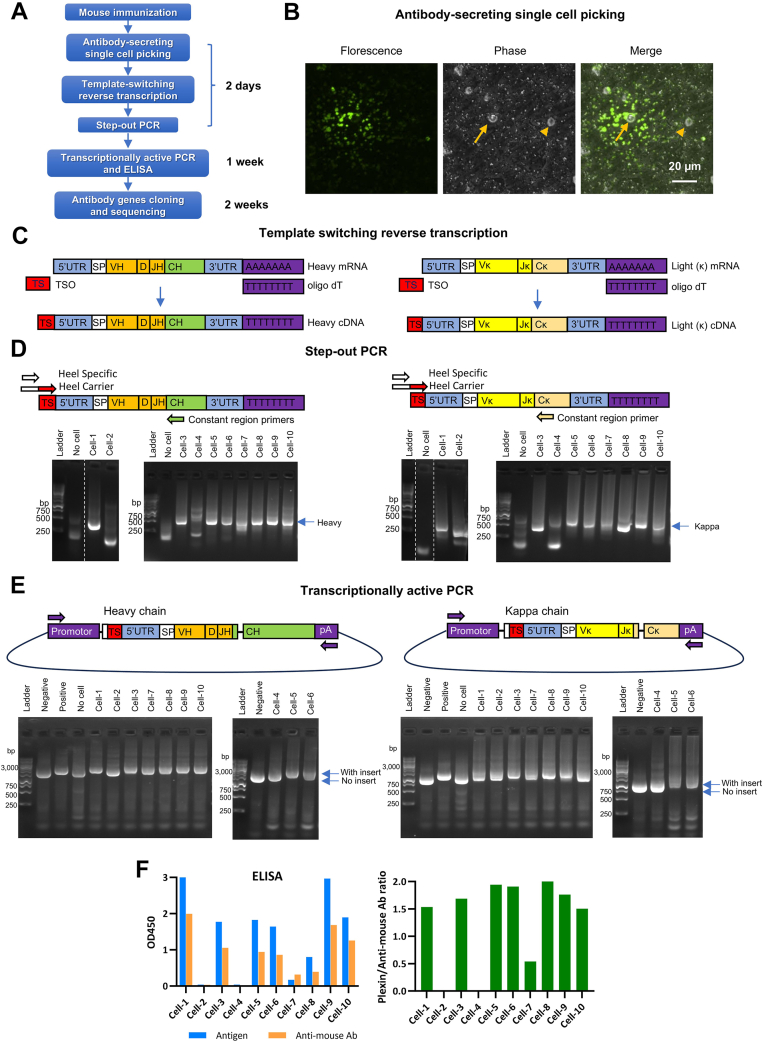
**Single B cell–based antibody discovery platform**. *A*, schematic representation of the antibody discovery platform workflow. *B*, fluorescent foci assay for identifying antibody-secreting B cells. *Arrow*: antibody-secreting B cell. *Arrowhead*: single cell not secreting the desired antibody. *C*, schematic illustrating template-switching reverse transcription for cDNA synthesis. *D*, schematic depicting step-out PCR for cloning heavy and kappa chain variable regions. Agarose gels show amplified heavy and kappa chain variable regions from step-out PCR. DNA bands at ∼500 bp indicate successful amplification. Samples were run on multiple agarose gels. Lanes from the same original gel were grouped together and *dotted lines* indicate lanes stitched. *E*, schematic illustrating transcriptionally active PCR for fusing promoter, constant regions, and polyA signal to enable antibody expression. Agarose gels show amplification products from transcriptionally active PCR. An upward shift of DNA bands on the agarose gel indicates successful fusion. *F*, antibodies expressed in culture supernatant were validate for antigen binding using ELISA.

The mAbs targeting extracellular domains (ECDs) are required to develop bsAbs-targeting NRP2 and PLXNA1. In a previous study, we developed and characterized a panel of aNRP2 mAbs targeting different NRP2 ECDs (42), whereas a diversified pool of aPLXNA1 mAbs were yet to be discovered. To achieve this goal, a state-of-the-art and highly efficient mAb discovery platform was established by combining techniques including CpG-DNA–containing adjuvant (50), fluorescent foci assay for cell picking (51, 52), template-switching reverse transcription (TS-RT) (53), step-out PCR (SO-PCR) (54), and transcriptionally active PCR (55) (Fig. 2*A*).

The Magic Mouse Adjuvant, which is a commercial CpG-DNA–containing adjuvant, was used to immunize mice as indicated in Materials and methods. To identify antibody-secreting cells, a fluorescent foci assay was used (Fig. 2*B*). In this assay, mouse splenocytes were mixed with antigen-coated beads and a fluorescent secondary antibody against mouse IgG and plate onto culture dish. After settling down to dish bottom, the antigen-coated beads immobilized and enriched antibody secreted by nearby mouse single cells. In the meanwhile, the secondary antibody made the immobilized antibody on the antigen-coated beads visible under fluorescence microscopy. Consequently, single B cells that secrete antibody can be identified by a fluorescent halo surrounding the cell of interest (Fig. 2*B*). The fluorescence-positive antibody-secreting single cells were picked up and transferred to PCR tubes.

TS-RT was carried out by using a reverse transcriptase with terminal transferase activity, which adds dCdCdC DNA sequence at the 3′-end of cDNA in the absence of a template (53). In the presence of a template-switching oligo (TSO), the reverse transcriptase incorporated the TSO sequence at the 3′ of cDNA (Fig. 2*C*). This TSO sequence would serve as the forward primer binding site for antibody gene cloning by SO-PCR.

SO-PCR was used to clone variable regions of antibody genes (54). The forward primer set, including heel carrier and heel specific primers, bound to the TSO region of cDNA. The reverse primers bound to the constant regions of antibody genes (Fig. 2*D*). Only mouse kappa light chains (LCs) but not lambda LCs were cloned in our protocol, as majority of mouse IgGs (∼95%) contain kappa LCs (56). In this test run, eight pairs of heavy and kappa chains were successfully cloned from 10 mouse single cells (Fig. 2*D*).

After cloning of paired heavy and LCs of an antibody, promoter, antibody constant regions, and polyA signal were fused to antibody variable regions by PCR, making the resultant PCR fragment transcriptionally active (Fig. 2*E*). The PCR fragment containing the antibody variable region gene exhibited an upward shift compared to the negative controls (Fig. 2*E*). Transfection of the transcriptionally active PCR fragments enabled Expi293F cells to express and secrete antibody into culture medium. The culture medium was used directly in ELISA to verify antibody binding (Fig. 2*F*). In this test run, eight antibodies were discovered to bind to the antigen. These antibody genes were cloned into a plasmid vector and analyzed by sequencing (Fig. 2*A*).

### Discovery of aPLXNA1 mAbs using the single B cell–based discovery platform

**Figure 3:**
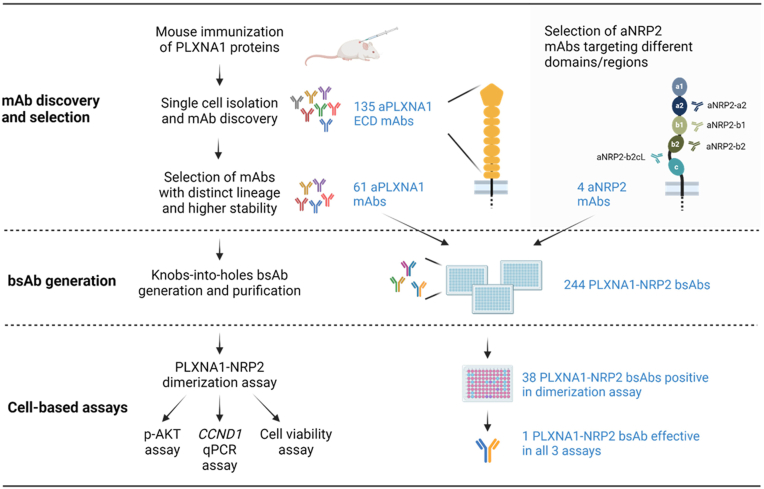
**Experimental workflow for the discovery of SEMA3F-mimetic bsAbs**. MAb-targeting PLXNA1 (aPLXNA1) were generated and paired with anti-NRP2 antibodies to create bispecific antibodies (bsAbs). These bsAbs were initially evaluated in a PLXNA1-NRP2 dimerization assay to identify candidates that effectively promote PLXNA1–NRP2 interaction. Positive bsAbs were then assessed using a p-AKT assay, a qPCR assay for *CCND1* expression, and a cell viability assay to determine which candidates mimic SEMA3F’s effects. Ultimately, one bsAb, P1943-Nb2cL, was identified as mimicking SEMA3F’s effects across all cell-based assays. aPLXNA1, anti-PLXNA1; NRP, neuropilin; PLXNA1, plexinA1; SEMA, semaphorin; p-AKT, phosphorylation of AKT; qPCR, quantitative PCR.

**Figure 4:**
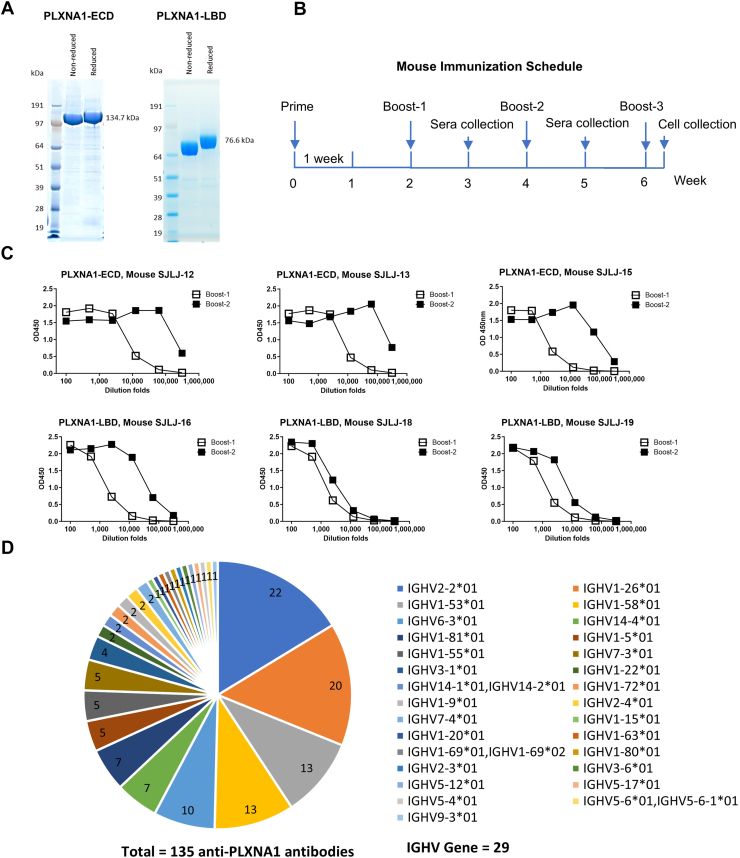
**Discovery of anti-PLXNA1 mAbs**. *A*, SDS-PAGE analysis of purified PLXNA1-ECD and PLXNA1-LBD proteins used for mouse immunization. The gel displays nonreduced (without reducing agent in the loading buffer) and reduced (with DTT added to the loading buffer) conditions. *B*, schematic illustrating the mouse immunization schedule, detailing the timeline for immunization, serum collection, and cell harvest. *C*, ELISA-based quantification of serum antibody levels following booster immunizations. Data represents six mice immunized with either PLXNA1-ECD or PLXNA1-LBD proteins. *D*, analysis of germline heavy chain IGHV gene usage in anti-PLXNA1 antibodies. A total of 135 cloned antibodies were analyzed, revealing usage of 29 distinct heavy chain IGHV genes. The *pie chart* shows the distribution of antibodies based on their IGHV gene usage. ECD, extracellular domain; LBD, ligand-binding domain; PLXNA1, plexinA1; SEMA, semaphorin.

The SEMA3F-mimetic bsAbs were developed and screened as described in Figure 3. First, aPLXNA1 (aPLXNA1) mAbs were generated utilizing the single B cell–based mAb discovery platform described above. Two recombinant human PLXNA1 proteins, full ECDs (27–1244 aa) and ligand-binding domains (LBDs, 27–710 aa), were purified (Fig. 4*A*). To produce B cells that secrete aPLXNA1 mAbs in mice, three SJL/J mice were immunized with each recombinant PLXNA1 protein using an immunization schedule spanning around 6 weeks (Fig. 4*B*). As shown in Figure 4*C*, mouse serum antibody titers increased by 2.3- to 60.9-fold after the second boost compared with the first boost, implying immunized mice successfully produced B cells–secreting aPLXNA1 mAbs.

In total, 248 single cells were picked from 6 mice, and 155 pairs of heavy and LCs were successfully cloned (Table 1 and Fig. S2). Among these 155 mAbs, 135 bound to PLXNA1 ECD in ELISA assay (Table 1 and Fig. S3). The average antibody cloning success rate (ELISA positive/picked single cells) was 53%, with success rates in individual mice ranging from 43 to 62%. After sequencing of the 135 aPLXNA1 mAbs, germline IGHV gene usage of the heavy chains (HCs) was analyzed. A total of 29 unique IGHV genes were discovered, with IGHV usage by individual mice ranging from 4 to 14 IGHV genes (Fig. 4*D* and Fig. S4).

**Table 1:** Summary of anti-human PLXNA1 antibodies discovery

No.	Mouse name	Antigen	Picked single cells	Paired heavy-light chains	ELISA positive	Selected antibodies
1	SJLJ-12	hPLXNA1 ECD	49	22	21	9
2	SJLJ-13	hPLXNA1 ECD	50	34	29	15
3	SJLJ-15	hPLXNA1 ECD	49	33	29	16
4	SJLJ-16	hPLXNA1 LBD	50	37	31	11
5	SJLJ-18	hPLXNA1 LBD	50	5	0	0
6	SJLJ-19	hPLXNA1 LBD	50	29	25	10
		Total (no SJLJ-18)	248	155	135	61

PLXNA1, plexinA1.

Of the 135 aPLXNA1 mAbs cloned, only a subset of mAbs was chosen to produce bsAbs using two criteria. First, only one aPLXNA1 mAb was selected from mAbs of the same mouse with the identical HC complementarity-determining region 3 (CDR3) sequence, as mAbs with the same CDR3 sequence likely share the same source B cell clone and bind to the same epitope (57). Second, mAbs with low stability motifs in CDR regions, including deamidation and isomerization motifs, were excluded (58). As a result, 61 aPLXNA1 mAbs were selected to pair with aNRP2 mAbs (Table 1, and Fig. 3).

### Selection of aNRP2 mAbs and generation of PLXNA1-NRP2 bsAbs

aNRP2 mAbs were generated by traditional hybridoma fusion method as previously described (42). Four high affinity aNRP2 mAbs that target distinct domains or region of NRP2 (aNRP2-a2, aNRP2-b1, aNRP2-b2, and aNRP2-b2cL) were selected based on the characterizations of epitope, binding affinity, specificity to NRP2, as well as the ligand blocking profile (Fig. 3, Table 2 and Fig. S5). These four aNRP2 mAbs bind to the a2, b1, b2, or linker between b2 and c (b2cL) domains of NRP2, respectively, with nanomolar to subnanomolar affinity (Table 2). aNRP2-b1 and aNRP2-a2 inhibit NRP2 binding to VEGFs and SEMA3F, respectively (42). aNRP2-b2 blocks both VEGFC and SEMA3F binding to NRP2, while aNRP2-b2cL does not interfere with ligand-induced NRP2–VEGFC and NRP2–SEMA3F interactions (Table 2).

**Table 2:** Characterization of aNRP2 mAbs

Anti-NRP2 name	Epitope domain on hNRP2^a^	Affinity to hNRP2_ECD^b^ (nM)	EC_50_ of binding to Expi293F-hNRP2 cells^c^ (nM)	Blocking of SEMA3F-induced NRP2/PLXNA1 dimerization^d^	Blocking of VEGF-induced NRP2/FLT4 dimerization^d^
aNRP2-b2cL	Between b2 and c	0.33 ± 0.04	n.d.	Partial	No
aNRP2-b1	b1	1.95 ± 0.2	0.33 ± 0.11	No	Strong
aNRP2-b2	b2	1.04 ± 0.04	2.70 ± 0.85	Strong	Strong
aNRP2-a2	a2	20.4 ± 2.2	1.75 ± 0.87	Strong	No

BLI, biolayer interferometry; ECD, extracellular domain; PLXNA1, plexinA1; NRP2, neuropilin 2; SEMA, semaphorin; VEGF, vascular endothelial growth factor.

a Epitope mapping was performed by ELISA and surface plasmon resonance (SPR) to various domains of hNRP2 ECD.

b Affinity was measured by BLI and shown as K_D_ values. Please note these antibodies were prior to affinity maturation and have different affinities from the affinity-matured antibodies described in (41). n.d. indicates not determined.

c The binding of aNRP2s to Expi293F-hNRP2 cells was analyzed by flow cytometry.

d Ligand-induced receptor dimerization was measured by the luciferase complementation assay. "Strong" indicates >90% blocking of ligand-induced receptor dimerization, "partial" indicates <80% inhibition at plateau, and "no" indicates < 10% inhibition. All these experiments were performed as previously described (41). Values shown are mean ± SEM from at least 3 independent experiments.

**Figure 5:**
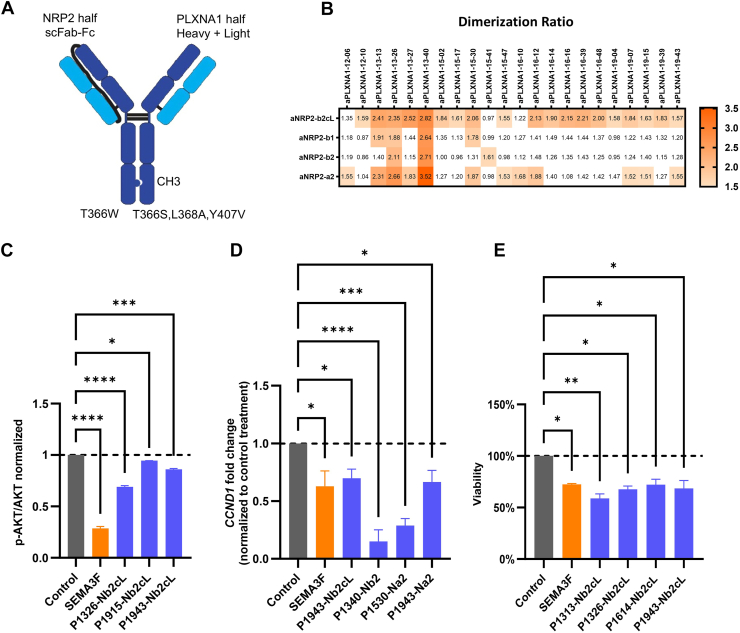
**Identification of PLXNA1-NRP2 bsAb with SEMA3F-mimicking mechanism and activities**. *A*, schematic illustrating structural format of PLXNA1-NRP2 bsAbs. The bsAbs are based on a human IGG4 backbone, with the light chain and heavy chain of the anti-NRP2 half linked by a 34 amino acid GS flexible peptide linker. The anti-NRP2 moiety contains a knob mutation, while the anti-PLXNA1 moiety features a hole mutation. *B*, screening for bsAbs that induce dimerization of cell surface PLXNA1 and NRP2. Each table cell represents a unique PLXNA1-NRP2 bsAb. bsAbs inducing a dimerization ratio change greater than 1.5 are considered positive hits and highlighted in *orange*. *C*, PLXNA1-NRP2 bsAbs that significantly reduced p-AKT level. *D*, PLXNA1-NRP2 bsAbs that significantly reduced *CCND1* expressing in qPCR assay. *E*, PLXNA1-NRP2 bsAbs that significantly inhibited proliferation of U-251 MG cells. All data are presented as the mean ± SEM from three experiments. aPLXNA1, anti-PLXNA1; bsAb, bispecific antibody; NRP, neuropilin; p-AKT, phosphorylation of AKT; PLXNA1, plexinA1; qPCR, quantitative PCR; SEMA, semaphorin.

A total of 244 PLXNA1-NRP2 bsAbs were generated by combining 61 aPLXNA1 with 4 aNRP2 mAbs (Fig. 3). The aPLXNA1 half of the antibody was expressed as independent HC and LC, which were directly derived from the antibody cloning platform, eliminating the need for subcloning or modification. The aNRP2 half was formatted into a single-chain Fab (scFab) instead of separate heavy and LCs to avoid mispairing with those of aPLXNA1. The scFab was oriented as LC-linker-HC to preserve the standard HC format of variable heavy-constant heavy 1 (CH1)-hinge-CH2-CH3, attaching the variable light kappa (VL-k) to the N terminus of the HC through the GS linker. To ensure accurate heavy-HC heterodimerization of aPLXNA1 and aNRP2 halves, knobs-into-holes mutations were applied to the aPLXNA1 (holes) and aNRP2 (knobs) components (Fig. 5*A*).

### Identification of PLXNA1-NRP2 bsAbs that mimic SEMA3F in cellular activities

A PLXNA1-NRP2 dimerization assay was used as a primary screen to identify PLXNA1-NRP2 bsAbs that could bring PLXNA1 and NRP2 together (Figs. 1*E* and 3). A representative PLXNA1-NRP2 dimerization assay is shown in Figure S6, *A* and *B*. Fold changes equal to or greater than 1.5 were considered as positive hits. This dimerization screening identified 38 positive PLXNA1-NRP2 bsAbs (Fig. 5*B*). These bsAbs were designated with the prefix "P" followed by the clone number of aPLXNA1 component, and the prefix "N" followed by clone number of aNRP2 component. It was found that the majority of bsAbs that facilitated PLXNA1/NRP2 dimerization were targeting a2 domain (SEMA3F-binding domain) or the flexible linker between b2 and c domain, and only a few bound b1 or b2 domain of NRP2 (Fig. 5*B*).

To identify SEMA3F-mimetic PLXNA1-NRP2 bsAbs with anticancer activities, three cell-based assays using U-251 MG cells—including p-AKT, *CCND1* oncogene expression, and cell proliferation assays—were employed to evaluate the 38 PLXNA1-NRP2 bsAbs identified in the dimerization assay (Fig. 3). In the p-AKT assay, we identified three PLXNA1-NRP2 bsAbs that mimicked SEMA3F to reduce p-AKT level (Fig. 5*C*). We also screened PLXNA1-NRP2 bsAbs in the qPCR assay for the *CCND1* reduction and identified four bsAbs that reduced its gene expression (Fig. 5*D*). Finally, in cell viability screening, four PLXNA1-NRP2 bsAbs reduced the viability of U-251 MG cells (Fig. 5*E*). The overall cell-based assay results were summarized in Table S5, with the bsAbs grouped based on their aNRP2 epitope. One bsAb, P1943-Nb2cL, mimicked SEMA3F’s effects in all the cell-based assays, was discovered and thus further investigated in structural studies to understand its binding sites on target receptors.

### SEMA3F-mimetic bsAb binds at distinct sites from SEMA3F to dimerize PLXNA1 and NRP2

**Figure 6:**
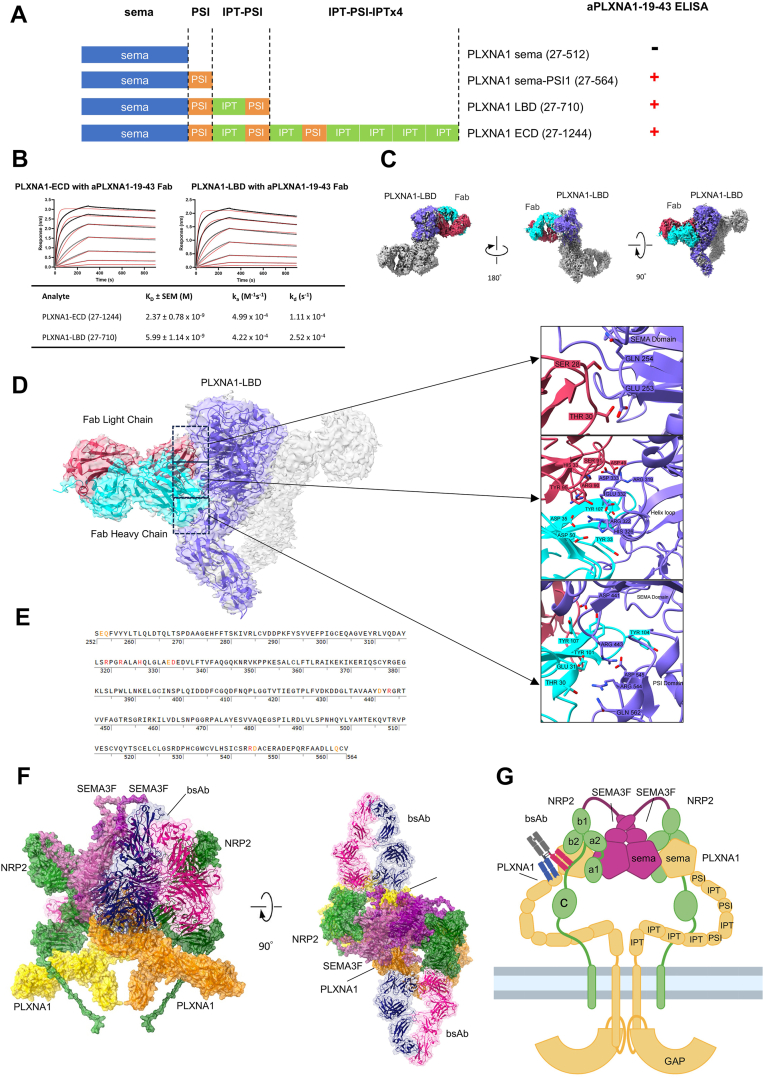
**Characterization of the binding mechanism of aPLXNA1-19-43 Fab to PLXNA1**. *A*, epitope mapping of aPLXNA1-19-43 Fab to PLXNA1. ELISA was used to evaluate binding of aPLXNA1-19-43 Fab to various PLXNA1 fragments. Results are indicated by ELISA readings, with positive (+) binding shown in *red* for readings >0.1 and negative (−) binding for readings <0.1. *B*, determination of binding affinity of aPLXNA1-19-43 Fab to PLXNA1 ECD and LBD. Representative sensorgrams from triplicate experiments are shown. The Fab was immobilized using biosensor tips coated with anti-mouse kappa antibody and subsequently exposed to a concentration series of PLXNA1-ECD or PLXNA1-LBD (*black and gray lines*). Data were fitted to a 1:1 binding model (*red lines*) to calculate binding constants. *C*, cryo-EM density map of the aPLXNA1-19-43 Fab in complex with PLXNA1-LBD. The 2:2 dimeric complex is displayed, with one subunit colored as follows: PLXNA1-LBD in *blue*, Fab heavy chain in *cyan*, and Fab light chain in *red*. *D*, schematic binding model of aPLXNA1-19-43 Fab with PLXNA1. The critical residues involved in binding are enlarged in the inserted *panels*. *E*, identification of critical residues on PLXNA1 that are involved in the binding of aPLXNA1-19-43 Fab. The Fab is shown to bind to both the SEMA and PSI domains of PLXNA1, with key interactions highlighted. *F*, binding model of P1943-Nb2cL to the PLXNA1–NRP2–SEMA3F complex. *G*, schematic representation of the binding mechanism between P1943-Nb2cL and the PLXNA1–NRP2–SEMA3F complex, providing a simplified visual overview. aPLXNA1, anti-PLXNA1; ECD, extracellular domain; Fab, fragment of antigen binding; LBD, ligand-binding domain; NRP, neuropilin; PLXNA1, plexinA1; PSI, plexin-semaphorin-integrin; SEMA, semaphorin.

To investigate the binding sites of bsAb P1943-Nb2cL to PLXNA1, we first analyzed the binding between the fragment of antigen binding (Fab) of the mAb aPLXNA1-19-43 and each of the four fragments of PLXNA1-ECD by ELISA (Fig. 6*A*). The minimal binding region for the aPLXNA1-19-43 Fab on PLXNA1 was determined to be the SEMA-PSI domain. The binding affinities of the aPLXNA1-19-43 Fab to PLXNA1-ECD and PLXNA1-LBD were 2.37 ± 0.78 nM and 5.99 ± 1.14 nM, respectively (Fig. 6*B*). Next, we purified the protein complex of PLXNA1-LBD and aPLXNA1-19-43 Fab and resolved its structure at a 3.33 Å resolution using cryo-EM (Fig. 6, *C*–*E*, Fig. S7 and Table S6). The structural analysis revealed a 2:2 complex formation between PLXNA1 and the Fab, wherein two SEMA domains of PLXNA1 dimerize the complex (Fig. 6*C*). This dimerization buries a surface area of 1562 Å^2^ between the two SEMA domains, as calculated by PDBePISA. The dimerization interface is characterized by two significant interactions: a π–π interaction between the side chains of His154 from each sema domain, and a polar interaction between the side chain of Arg142 of one SEMA domain and the backbone carbonyl of Pro88 of the other (Fig. 6, *D* and *E*). These binding sites on the aPLXNA1-19-43 Fab are distinct from the SEMA3F-binding region located at the SEMA domain.

To assess the specificity of the bsAb P1943-Nb2cL binding to PLXNA1 and NRP2, we first performed ELISA assays with ECDs of various PLXNs and NRPs. The results showed that bsAb P1943-Nb2cL binds specifically to PLXNA1 and NRP2, but not to PLXNA2-3, PLXNB1, PLXND1, or NRP1 (Fig. S8*A*). We further performed a dimerization assay using Expi293F cells expressing various pairs of NRPs-Lg and PLXNAs-Sm. The cells were treated with bsAb P1943-Nb2cL at 100 nM, which specifically induced the dimerization of the PLXNA1-NRP2 pair but no other PLXN-NRP pairs (Fig. S8*B*). Additionally, protein sequence alignments among PLXN proteins (Fig. S8*C*) revealed that other PLXNs lack critical residues required for the bsAb binding (Fig. 6), which is consistent with the ELISA and dimerization assay results. Collectively, these findings demonstrate that bsAb P1943-Nb2cL is specific in binding and dimerization of NRP2 and PLXNA1.

To explore the structural basis for the bsAb activation of the PLXNA1-NRP2 receptors, we built a structural model based on the aPLXNA1-19-43 Fab and PLXNA1 complex structure as well as aNRP2-b2cL binding site on NRP2. Published structure of PLXNA4–NRP1–SEMA3A complex was used as template (47) (Fig. 6, *F* and *G*). This model shows that the bsAb P1943-Nb2cl binds to the linker between b2 and c domain of NRP2 (amino acids 595–650), and sema and PSI domains of PLXNA1. These binding sites of P1943-Nb2cL are distinct from those of SEMA3F, which targets a1 and a2 domain of NRP2 (46, 59), and SEMA domain of PLXNA1 (60). The b2c linker of NRP2 is flexible and in space proximity to the SEMA-PSI domain of PLXNA1 in the SEMA3F-bound conformation, with the minimal distance at 36 Å (Fig. 6*G*). The two Fab arms of an antibody bind bivalently to two antigens separated at distances ranging from 3 to 17 nm (61). Therefore, the binding of P1943-Nb2cL bsAb to NRP2 and PLXNA1 allows proper spacing of the two receptors similarly to the natural ligand SEMA3F. Furthermore, the binding site on NRP2 is a flexible loop allowing flexibility of conformational changes of the receptor complex for downstream signaling. Indeed, the proximity of PLXNA1 and NRP2 was not sufficient to exert agonist effects, as evidenced by the other bsAbs that could dimerize the receptors but not exhibit consistent SEMA3F-like activities in the cell assays. This indicates that the bsAb P1943-Nb2cL binding induced an allosteric effect that resulted in desired conformational changes in the receptor complex to trigger downstream signaling.

## Discussion

SEMA3F has been extensively studied for its anticancer activities. However, few studies directly used SEMA3F as a therapeutic agent (25), likely due to its unfavorable drug properties. Supporting this notion, we found that SEMA3F-p65, a furin-cleaved SEMA3F, loses its ability to inhibit the proliferation of U-251 MG cells (Fig. 1, *B* and *C*). Given that furin is ubiquitously expressed and present on the membranes of mammalian cells (62), our results suggest that SEMA3F would be unstable in human body, resulting in a short half-life and low bioavailability. However, these issues can be addressed by SEMA3F-mimetic bsAbs by offering better pharmacological properties, including enhanced stability and a longer half-life (63). The current study developed and characterized P1943-Nb2cL as the first agonist bsAb that mimics anticancer activities of SEMA3F, demonstrating the feasibility of developing PLXN-NRP bsAbs to mimic class 3 SEMAs. Class 3 SEMAs and their receptors, PLXNs and NRPs, are implicated not only in tumor progression (40, 64) but also in angiogenesis (65), metabolic disorders (66), and allergic diseases (67). Development of SEMA-mimetic bsAbs targeting these diseases may position PLXN-NRP bsAbs as a promising novel class of biologics for drug development.

In this study, an efficient antibody discovery platform based on single-cell technology has allowed the discovery of more than 60 diversified aPLXNA1 mAbs and facilitated the generation of SEMA3F-mimetic bsAbs. This platform integrates multiple advanced technologies for antibody cloning, resulting in a novel platform not previously reported. First, the fluorescent foci assay (51, 52) combined with CellCelector or laser microdissection microscope is an effective approach for antibody-secreting single-cell identification and isolation. The template switching reverse transcription (53) and SO-PCR (54) offer significant advantages in efficiency and specificity of antibody gene cloning. Additionally, transcriptionally active PCR (55) enables the rapid screening of cloned antibodies without the need for plasmid construction, sequencing, or gene synthesis, which reduces both time and cost for antibody discovery. The overall success rate of this antibody discovery platform, defined as the ratio of paired heavy-light genes to picked single cells, ranges from 45 to 74% for individual mice, with an average success rate of 63%. This rate is comparable to or exceeds that of other published platforms (52, 68, 69, 70, 71, 72). The antibody cloning platform demonstrates high diversity, with 4 to 14 HC V-genes cloned from individual mice, indicating its ability to capture most antibody species. Notably, up to 14 HC V-genes were cloned from the mouse SJLJ-15 (Fig. S4*C*), with 10 of them appearing only once, highlighting the platform’s ability to identify rare mAbs. This platform can clone mAbs from diverse B cell sources across multiple species, including bone marrow, lymph nodes, or peripheral blood mononuclear cells from rabbits, llamas, humans, or other species. By modifying the cell identification method, this platform can clone T-cell receptors. Additionally, the cloning method reported in this study is fully compatible with fluorescence-activated cell sorting–based single B cell sorting for mAb cloning.

In the PLXNA1-NRP2 dimerization assay, luciferase tagged at different positions affected the magnitude of dimerization ratio changes by SEMA3F treatment (Fig. S1, *A*–*D*). The N-terminal luciferase tag induced a larger ratio change than the C-terminal luciferase tag (N-terminal ratio 4.3 *versus* C-terminal ratio 1.5). This was probably due to the difference in distance between two split luciferase portions. Upon SEMA3F treatment, the N-terminal luciferase portions may get closer than the C-terminal luciferase split tags. This is supported by the model in Figure 6*F* that N termini of PLXNA1 and NRP2 are close enough to bring split luciferase parts together when the SEMA3F–PLXNA1–NRP2 complex forms. In contrast, C termini of PLXNA1 and NRP2 may be too far apart to bring split luciferase together. Longer distance between C termini of PLXNA1 and NRP2 implies that the NRP2 C-terminal intracellular sequence may not be involved in signaling transduction by PLXNA1.

It has been reported that in the absence of SEMA, PLXNA1 exists in an autoinhibitory state and the SEMA domain of PLXNA1 mediates this autoinhibition by interacting with PSI2-IPT2 domains of another PLXNA1 in a “head-to-stalk” manner (73, 74). Interestingly, our structural study shows that two PLXNA1 proteins form a new type of homodimer by a “head-to-head” interaction, in which two SEMA domains mediate homodimerization (Fig. 6, *C* and *D* and Fig. S9, *A*–*C*). This SEMA–SEMA homodimer interface is different from the previously reported SEMA–SEMA interface of PLXNA2 (60). We propose that this homodimerization of PLXNA1 may represent another autoinhibition mechanism of PLXNA1 (Fig. S9*D*). Structural analysis revealed that this head-to-head interaction separates PLXNA1 intracellular GTPase-activating protein domain far apart enough to prevent dimerization and activation of PLXNA1 (Fig. S9*D*).

BsAbs have emerged as a leading drug class for cancer therapy and other therapeutic applications (75, 76). More than 100 bsAbs are under clinical evaluation, and the majority fall into categories of bispecific immune cell engager, those targeting two tumor-associated antigens, and those blocking signaling pathways (75, 77). However, to our knowledge, no agonistic bsAb is currently being evaluated in clinical trials, although there have been several reports of ligand-mimetic bsAbs at the stage of research or preclinical studies. These include bsAbs as factor VIII-mimetics with hemostatic or cofactor activities (78, 79), FGF21-mimetics with metabolic activities (80, 81), IL-2-mimetics that promote expansion of lymphocytes (82, 83), a type-I IFN-mimetic with antiviral ability (83), an EPO-mimetic to regulate red blood cell production (84), and an IL-18-mimetic for cancer therapy (85). The difficulty in developing ligand-mimetic bsAbs may lie in that such bsAbs are required to not only bring two target proteins together to facilitate complex formation but also induce proper conformational changes of target protein(s) to activate downstream signaling. We found P1943-Nb2cL binds to non–ligand-binding regions on target receptors, which was also reported for the FGF21-mimetic bsAb (80). These findings and our structural model suggest that the ligand-mimetic bsAbs may not share binding sites with natural ligands. Instead, it is more important that the binding sites of two bsAb Fabs allow proper spacing for receptor complex formation in a favorable conformation similar to that induced by the ligand binding, as well as sufficient flexibility of conformational changes for downstream signaling. These provide insights for molecular designing of agonistic bsAbs, for example, using computational methods to predict optimal binding regions.

Based on this study, the SEMA3F-mimetic bsAb P1943-Nb2cL shows promise for preclinical mouse models and eventually clinical trials targeting glioblastoma and other cancers that express high levels of NRP2 and PLXNA1. As a bsAb, P1943-Nb2cL is expected to exhibit improved stability and a longer half-life than native SEMAs. This would allow less frequent dosing compared to a SEMA used as a therapeutic, and thus minimize stress and treatment burden in preclinical and clinical studies. Moreover, the bsAb specifically targets a tumor-suppressive pathway of SEMA3F, which may translate to improved efficacy and fewer side effects than SEMAs. One potential issue for further investigation of P1943-Nb2cL in glioblastoma models is the difficulty of antibody penetration across the blood-brain barrier to access the cancer cells, which may reduce the bsAb bioavailability and anticancer efficacy. This is a general limitation of biologic drugs, and various intracerebral drug delivery techniques are being explored for efficient brain delivery, including spinal injections, hydrogen gel (86), focused ultrasound, receptor-mediated transcytosis, and nanoparticles (87). Alternatively, P1943-Nb2cL may be evaluated in other types of tumors that highly express NRP2 and PLXNA1. For example, both NRP2 and PLXNA1 are significantly upregulated in renal cell carcinoma (the Human Protein Atlas, Fig. S10), which may be a promising target cancer type for therapeutic investigations of P1943-Nb2cL.

In summary, to develop SEMA3F-mimetic bsAb(s) as candidates for new anticancer therapy, we characterized SEMA3F signaling and activity in glioblastoma U-251 MG cells. Based on these findings, we screened over 200 PLXNA1/NRP2 bsAbs generated in this study and discovered P1943-Nb2cL that mimicked SEMA3F in cell-based assays, including dimerization of PLXNA1/NRP2, downregulation of phospho-AKT and *CCND1* gene expression, and inhibition of U-251 MG cancer cell proliferation. This study demonstrates the efficient generation and screening of novel bsAbs for therapeutic candidates using a single cell–based mAb discovery platform. Importantly, this work also provides structural insights that expand the greater understand of the molecular mechanisms underlying SEMA3F signaling through PXNA1/NRP2 and may facilitate the development of SEMA-mimetic bsAbs with anticancer potential as well as agonistic bsAbs for other therapeutic applications.

## Experimental procedures

### Recombinant proteins

The vector used was pNTC7485 from the Nature Technology Corporation. The vector was modified to append a secretory signal peptide (SPARC) to the expressed protein, which drives secretion into the cell media and is removed during protein translocation.

Purification of human SEMA3F is described in the previous paper (42). Briefly, a signal peptide from the SPARC gene (MRAWIFFLLCLAGRALA), a 6xHis-Myc tag (HHHHHHEQKLISEEDLG), and a GS linker (GGGGS) were fused to the N terminus of SEMA3F (XP_054203483.1, amino acids 19–748 with R582A and R585A) or SEMA3F-p65 (XP_054203480.1, amino acids 19–586). These DNAs were cloned into the mammalian expression vector. Resultant plasmids were transfected into Expi293F cells cultured in Expi293 Expression Medium (Thermo Fisher Scientific, Cat#A1435102) using the ExpiFectamine 293 Transfection Kit (Thermo Fisher Scientific, Cat# A14524). After 5 days of expression, the culture medium containing the protein was filtered with 0.22 μm filter, and the proteins were purified with a HiTrap MabSelect column (Cytiva).

For expression and purification of PLXNA1 proteins, DNA encoding human PLXNA1-ECD (NP_115618.3, amino acids 1–1244) or PLXNA1-LBD (NP_115618.3, amino acids 1–710) were fused with 6xHis tag at the C terminus and cloned into the mammalian expression vector. The plasmids were transfected into Expi293F cells and PLXNA1 proteins secreted in the culture media were purified with the HiTrap MabSelect column (Cytiva).

For Fab protein expression and purification, DNAs encoding Fab HC with a His-tagged at the C terminus and untagged LC (both in mouse IgG1 backbone) were cloned into the mammalian vector and transfected in Expi293F cells. Fab in culture medium was purified using the HiTrap MabSelect column (Cytiva).

For the purification of the PLXNA1 and aPLXNA1 Fab–P1943 complex, the purified PLXNA1-LBD and aPLXNA1 Fab were mixed in a 1:1.3 M ratio in a buffer containing 20 mM sodium acetate, pH 5.0. The protein mixture was loaded in 2 cycles onto a 0.68 ml POROS XS column equilibrated with 20 mM sodium acetate, pH 5.0. The column was then washed with EQ buffer (50 mM Tris–HCl, 0.5 M NaCl, pH 7.4). Excess aPLXNA1 Fab was washed off the column with 25% buffer B (buffer B: 20 mM sodium acetate, 1 M NaCl, pH 5.0) in EQ buffer. The PLXNA1–Fab complex was eluted with 60% buffer B. Main peak fractions were pooled and concentrated using a 10 kDa molecular weight cut-off centrifugal protein concentrator prior to loading onto a 120 ml Superdex 200 size exclusion chromatography column equilibrated with 20 mM Tris–HCl, 150 mM NaCl, pH 7.4. Main peak fractions from the Superdex were concentrated with a 10 kDa molecular weight cut-off centrifugal protein concentrator, filtered with 0.2 μm centrifugal polyvinylidene difluoride filters, and stored at −80 ^o^C.

For bsAb purification, plasmids encoding aNRP2 scFab-Fc as well as aPLXNA1 antibody heavy and kappa chains were cotransfected into ExpiCHO-S cells using the ExpiFectamine CHO Transfection Kit (Thermo Fisher Scientific, Cat# A29133). The sequences of the variable domains for constructing the bsAb P1943-Nb2cL are shown in Table S7. After 5 days of expression, the bsAb in the culture medium was purified using a Protein G HP SpinTrap column (Cytiva, 28903134).

### Mouse immunization, titration of serum antibodies, and single-cell collection

Mice immunization experiment in this study was conducted in compliance with the protocol approved by the Animal Ethics Committee at the Hong Kong University of Science and Technology. SJL/J mice were purchased from The Jackson Laboratory (Strain: 000686) and maintained in a 12-h light/dark cycle with free access to water and food. Mice were first primed with 1 × 10^6^ Expi293F cells expressing full-length human PLXNA1 followed by 2 boosts with purified human PLXNA1 proteins in Magic Mouse Adjuvant (Creative Diagnostics # CDN-A001) according to supplier’s instruction. One week after boost, blood was collected from mouse tail vein and serum antibody was titrated by ELISA.

To determine the titer of antibodies in the serum of immunized mice, specific antigens, either PLXNA1-ECD or PLXNA1-LBD, were coated onto an ELISA plate and allowed to incubate overnight at 4 °C. The following day, mouse sera were diluted in SuperBlock blocking buffer (Thermo Fisher Scientific, Cat#37515) to a series of concentrations: 1:100, 1:500, 1:2500, 1:12,500, 1:62,500, and 1:312,500. A volume of 100 μl of each diluted serum sample was then added to the corresponding wells of the ELISA plate and incubated at room temperature for 1 h to allow for antibody-antigen binding. After incubation, the plates were washed to remove unbound serum components. Subsequently, 50 μl of a secondary antibody solution, consisting of goat anti-mouse IgG (H + L) horseradish peroxidase (Thermo Fisher Scientific, Cat# G-21040) diluted to a concentration of 0.2 μg/ml, was added to each well. The plates were then incubated for an additional 30 min at room temperature to facilitate the binding of the secondary antibody to the primary antibodies. Following a second round of washing, 50 μl of 3,3′,5,5′-tetramethylbenzidine substrate solution (Thermo Fisher Scientific, Cat# N301) was added to each well, and the plates were incubated at room temperature for 7 min to develop color. The enzymatic reaction was halted by the addition of 50 μl of 1 M HCl. The absorbance at 450 nm was measured using a microplate spectrophotometer (Thermo Fisher Scientific, Cat# A51119600C).

Splenocytes from individual mice were isolated and collected following a modified version of a previously established protocol (88). Mice were immunized with PLXNA1 proteins using Magic Mouse Adjuvant 3 days prior to cell collection. On the day of cell collection, the mice were humanely euthanized by cervical dislocation under strict adherence to ethical guidelines for animal research. The spleens were dissected and transferred to a sterile Petri dish containing 5 ml of Hank's Balanced Salt Solution (HBSS, Thermo Fisher Scientific, Cat# 14170161). Each spleen was then cut into 3 to 5 pieces using sterile scissors and forceps. The spleen tissue was placed onto a 70 μm cell strainer (Corning, Cat# 431751) and mashed using the plunger of a 3 ml syringe to release single splenocytes. To obtain a suspension of single cells, the crushed spleen was washed with HBSS twice to remove debris and undesired cell aggregates. The cells were collected and stored in a cryopreservation solution consisting of RPMI 1640 medium (Thermo Fisher Scientific, Cat#A1049101) supplemented with 10% fetal bovine serum (FBS; Thermo Fisher Scientific, Cat#10439024) and 10% dimethyl sulfoxide (Sigma-Aldrich, Cat#D2438-50 ML). The cell suspension was aliquoted into cryogenic vials and stored in liquid nitrogen for future use.

### Single B cell identification and picking

The identification of single B cells of interest was performed using a modified fluorescent foci method, as previously described (52). Splenocytes were rapidly thawed and washed once with RPMI 1640 medium (Thermo Fisher Scientific, Cat# A1049101) to remove cryoprotectants before proceeding to the fluorescent foci assay. A suspension of one million mouse splenocytes was prepared in RPMI 1640 medium and mixed with an Alexa Fluor 488–conjugated secondary antibody (Thermo Fisher Scientific, Cat# A11017) and Ni-NTA Magnetic Beads (Thermo Fisher Scientific, Cat# 88831) that had been preconjugated with the purified PLXNA1-ECD protein. This mixture was carefully pipetted into each well of a 6-well plate and incubated at 37 °C with 5% CO_2_ for 1 to 2 h to allow for the formation of fluorescent foci. Following the incubation period, cells surrounded by a fluorescent halo, indicative of successful antigen-antibody binding, were identified, aspirated and collected using automated cell picker (CellCelector, ALS) or laser microdissection microscope (PALM Microbeam, Zeiss). Each collected single cell was transferred into a 200-μl PCR tube containing a 7.21 μl lysis buffer composed of a 20-mer dT primer at a final concentration of 2 μM (Integrated DNA Technologies, Cat# 51-01-15-01), 0.04% Triton X-100 (Thermo Fisher Scientific, Cat# 28314), and 14 Units of RNaseOUT (Thermo Fisher Scientific, Cat# 10777019) to facilitate subsequent antibody gene cloning. The PCR tubes were stored at −80 °C to preserve the integrity of mRNAs for future gene cloning.

### Antibody gene cloning

TS-RT was performed on single cells stored in lysis buffer, following a modified protocol based on reference (53). Cells were rapidly thawed by heating at 72 °C for 3 min and then immediately placed on ice to prevent degradation. A 6.79 μl reaction mixture was prepared, containing a final concentration of 1 mM dNTPs, 2 mM DTT, 1x first-strand buffer, 5 U/μl SMARTScribe Reverse Transcriptase (TaKaRa, Cat# 639538), and 1 μM TSO (AAGCAGTGGTATCAACGCAGAGTACGCrGrGrG). Reverse transcription was carried out at 42 °C for 60 min, followed by enzyme inactivation at 70 °C for 15 min. The resulting cDNA was directly used for amplification of the antibody heavy and LCs.

SO-PCR was conducted to amplify the antibody HC and LC, as described in (54) with modifications. The heel carrier and heel-specific primer sequences were GTTACAGATCCAAGCTGTGACCAAGCAGTGGTATCAACGCAGAGT and GTTACAGATCCAAGCTGTGACC, respectively. Mouse heavy constant region-specific primers for Ighg1, Ighg2b, Ighg2c, and Ighg3 were GTCACTGTCACTGGCTCAGGGAAATAG, GTCACAGTCACTGACTCAGGGAAGTAG, GTCAAGGTCACTGGCTCAGGGAAATAA, and TTTACAGTTACCGGCTCAGGGAAGTAG, respectively. The mouse kappa constant region-specific primer was GTTCAAGAAGCACACGACTGAGGCAC. A 20 μl SO-PCR reaction mixture was prepared with 0.02 μM heel carrier primer, 0.15 μM heel-specific primer, and 0.15 μM constant region-specific primer. The thermal cycling program consisted of 15 cycles of 98 °C for 10 s, 71 °C for 30 s, and 72 °C for 60 s, followed by 35 cycles of 98 °C for 10 s, 62 °C for 30 s, and 72 °C for 30 s.

The SO-PCR products were cloned into plasmid vectors containing a promoter, either the heavy or kappa constant regions, and a polyA signal using the NEBuilder HiFi DNA Assembly Master Mix (New England Biolabs, Cat# E2621X). The transcriptionally active fragments were amplified by PCR and transfected into Expi293F cells (Thermo Fisher, Cat# A1435102) cultured in Expi293 Expression medium using the ExpiFectamine 293 Transfection Kit (Thermo Fisher Scientific, Cat# A14524). The culture media containing the expressed antibodies were screened for antibody presence using ELISA.

### NRP2 and PLXNA1 dimerization assay

Dimerization between cell surface NRP2 and PLXNA1 proteins were determined using a luciferase complementation assay as described previously (42). cDNAs of NRP2 and PLXNA1 were obtained from OriGene and R&D Systems respectively (NRP2 C220706, PLXNA1 RDC0967). The NRP2 was cloned into pBiT vector containing small fragment of the NanoLuc Luciferase (SmBiT, 1.3 kDa), while PLXNA1 was cloned into pBiT vector containing the large fragment of the NanoLuc Luciferase (LgBiT, 18 kDa). Both tags were positioned at the N-terminal, extracellular end of their respective receptors, and separated from the receptor by the vector encoded flexible linker. Plasmids were transfected at equal mass at 0.5 μg of each plasmid per 1 × 10^6^ per ml Expi293F cells using ExpiFectamine kit (Thermo Fisher Scientific) according to the manufacturer’s protocol. The transfected Expi293F was harvested after 16 to 18 h incubation under 8% CO_2_ at 37 ^o^C. The cells were then counted using CellCounter (Thermo Fisher Scientific) and plated in white luminometer plate at 50,000 cells per well in 80 μl Expi293 medium. After adding 20 μl of 1:20 dilution cell-permeable luciferase substrate (Nano-Glo Luciferase Assay System, Promega), baseline luminescence reading was recorded for 10 min using SpectraMax L microplate reader (Molecular Devices). Then, bispecific antibodies were added, and luminescence were recorded over time. Luminescence readings after treatment were first normalized to the baseline luminescence reading. The dimerization ratio was then calculated by dividing the normalized luminescence reading by the normalized reading of an untreated well at the same time point. The dimerization ratio for each bsAb was calculated by averaging the dimerization ratios measured within the first 20 min after antibody addition.

### Phospho-AKT assay

Human glioblastoma U-251 MG cells (Sigma Cat#09063001) were seeded at 100,000 cells per well in 1 ml Eagle’s Minimum Essential Medium (EMEM, ATCC, 30-2003) medium with 10% FBS (Thermo Fisher Scientific, 10439024) in a 24-well plate and incubated for 24 h at 37 °C with 5% CO_2_. After serum-free starvation for 20 h in 1 ml EMEM, cells were treated with bsAbs at 300 nM or 25 nM SEMA3F as a positive control for 30 min. After treatment, the plate was cooled down on ice for 10 min and the cells were washed twice using 1 ml cold HBSS buffer (Thermo Fisher Scientific, 14170112). For each well, cells were lysed and collected by using 250 μl 1x PBS containing 1x phosphatase, protease inhibitor (Thermo Fisher Scientific, A32959) and 0.5% Triton X-100 (Thermo Fisher Scientific, 28314). The cell lysate was then subjected to centrifugation at 14,000 rpm for 10 min at 4 °C from which supernatant was collected for assay measurement. Phospho-Akt (pS473) and total Akt were measured using the Akt (pS473)+total Akt ELISA Kit (abcam, ab126433). The Phospho-Akt over total Akt ratio was calculated by normalizing phospho-Akt (pS473) against total Akt.

### Real-time qPCR assays of PI3K-AKT and mTOR pathway genes

The U251 cells were seeded at 100,000 cells per well in 1 ml EMEM (ATCC, 30-2003) medium with 10% FBS (Thermo Fisher Scientific, 10439024) in a 24-well plate and incubated for 24 h at 37 °C with 5% CO_2_. After serum-free starvation in 500 μl EMEM overnight, the cells were treated with 25 nM SEMA3F diluted in media or media only as control. Cells were incubated at 37 °C for 1 or 6 h and then harvested. Total RNA was extracted using the RNeasy kit (Qiagen). The cDNA was synthesized using the iScript kit (Bio-Rad). RT2 Profiler PCR Arrays were ordered from Qiagen for profiling the human PI3K–AKT signaling pathway and mTOR signaling pathway (catalog number: PAHS-058Z and PAHS-098Z). Each array contains primers for 84 genes most relevant to the indicated pathway, and 5 house-keeping genes as well as other controls. The qPCR experiments were performed in a 384 plate on the ViiA7 real-time PCR system (Applied Biosystems) according to manufacturer’s instructions. For data analysis, NormFinder (89) was employed to identify the most stable house-keeping genes across samples, and two genes (*GAPDH* and *RPLR0*) were finally used for the normalization of expressions of target genes.

### qPCR screening assay for *CCND1* gene expression

The U251 cells were seeded at 50,000 cells per well in 500 μl EMEM with 10% FBS in a 48-well plate and incubated for 24 h at 37 °C with 5% CO_2_. After serum-free starvation for 20 h in 500 μl EMEM, the cells were treated for 8 h with 100 nM bsAbs or 30 nM SEMA3F as a positive control. The cells were then harvested and *CCND1* expression was quantified using FastLane Cell RT-PCR kit (Qiagen) according to manufacturer’s protocol. The qPCR primers for *CCND1*, beta-2-microglobulin and *GAPDH* genes were purchased from RT2 series of Qiagen in which primer pairs have been experimentally verified by manufacturer (GeneGlobe IDs: PPH00128F-200, PPH01094E-200, PPH00150F-200). The qPCR experiments were performed using the ViiA 7. Conditions of amplification were set following the suggested specifications for real-time one-step RT-PCR using cyclers from Applied Biosystems (Qiagen). This consists of a template denaturation at 95 °C for 15 min followed by a 40-cycle of amplification at 94 °C for 15 s, 60 °C for 30 s, and 72 °C for 30 s. *CCND1* expression was normalized by two reference genes beta-2-microglobulin and *GAPDH*. Experiments were performed in triplicate.

### Viability assay

Cells were seeded at 2500 cells per well in 100 μl EMEM (ATCC, 30-2003) medium with 10% FBS (Thermo Fisher Scientific, 10439024) in a 96-well white plate (Thermo Fisher Scientific, 136101) and incubated for 24 h at 37 °C with 5% CO_2_ for 24 h. After washing with serum-free EMEM, U251 cells were serum starved in 100 μl serum-free EMEM for 24 h. Serum-starved cells were subjected to a 72-h treatment of 300 nM bsAbs or 25 nM SEMA3F as a control. Cell viability of each sample was quantified using CellTiter-Glo 2.0 Assay (Promega) according to manufacturer’s protocol. Luminescent signal was measured using SpectraMax L microplate reader (Molecular Devices). Cell viability was normalized based on the nontreated control cells.

### Binding affinity measurements

Binding affinities of the aPLXNA1 Fab 19 to 43 for recombinant PLXNA1 ECD (aa 27–1244) or PLXNA1 LBD (aa 27–710) proteins were measured by biolayer interferometry on an Octet Red96e instrument (Sartorius). Biotinylated CaptureSelect anti-LC-kappa-murine antibody (Thermo Fisher Scientific) was immobilized on Octet streptavidin biosensor tips. The tips with anti-murine antibody were used to capture the aPLXN Fab and then dipped into solutions of either PLXNA1 ECD or PLXNA1 LBD at varying concentrations (1350, 450, 150, 50, 16.67, 5.56, 1.85 nM). The binding data for each protein was globally fitted to a 1:1 binding model in the Octet Data Analysis HT 11.1 software (https://www.sartorius.com/en/products/biolayer-interferometry/octet-systems-software) to obtain the binding constants.

### Electron microscopy

To prepare cryo-grids, 3 μl samples were applied to glow discharged Quantifoil Au grids (R2/2, 300 mesh), which were subsequently blotted with filter paper (Ted Pella) for 3 s at 18 °C and 100% humidity. The grids were immediately plunge frozen in liquid ethane using a FEI Vitrobot IV (Thermo Fisher Scientific). Quality of grids was screened using a FEI Titan Krios G3i electron microscope operated at 300 kV. Movies were collected from high quality grids using a FEI K3 Summit direct electron detector (Gatan), at a magnification of 81,000× and defocus range of −1.0 to −2.5 μm. A total of 40 frames were collected for each movie, with a total electron dose of 50 e/Å^2^.

### Image processing

Cryo-EM images were processed using CryoSPARC v4.1.2 (90) (Fig. S7). A total of 2084 movies were acquired and imported into Cryo-SPARC using the following parameters: Raw pixel size 1.026 Å, accelerating voltage 300 kV, spherical aberration 2.7 mm, and total exposure dose 50 e/Å^2^. Motion correction and contrast transfer function (CTF) estimation were performed using full-frame motion correction and patch CTF. After that, 1989 micrographs were selected from the 2084 micrographs based on average intensity (−10055.05–2041.77), CTF fit resolution (2.738–8.539 Å), defocus tilt angle (0.2–19.4 degrees), relative ice thickness (0.994–1.13), total full-frame motion distance (1.66–27.37 pixels), and full-frame motion curvature (0.83–5.58). Particles were picked by Blob Picker with the following parameters: minimum particle diameter 30 Å, maximum particle diameter 180 Å, minimum separation distance 0.5 diameter, and maximum number of local maxima to consider 10,000. After inspect particle picks (NCC score > 0.620, 10,421 < local power score < 17,132) and extract from micrographs (extraction box size 320 pix), a total of 3,741,859 particles were extracted. Junk particles were removed by three rounds of 2D classification and one round of 3D classification, which resulted in 1,047,987 high quality particles. These particles were classified into six 3D classes using *ab initio* reconstruction and heterogeneous refinement. Symmetry-free nonuniform refinement was performed with 253,734 particles from the largest class, resulting in a density map with 3.33 Å resolution. Local resolution of the density map was estimated by local resolution estimation (Fig. S7).

### Model building

Model building was initiated using rigid body docking of the crystal structure of PLXNA1 ECDs (PDB ID: 7y4p) (91) and AlphaFold (92) predicted model of the aPLXNA1 mAb Fab using molecular visualization program ChimeraX (93). To optimize fitting of the structures into the map, manual fitting using COOT was employed (94) and refinement through the real-space refinement module of Phenix (95). Default settings on real-space refinement module were used to improve the geometry and clash score of the model. The final model only included the SEMA and PSI1 domain of PLXNA1 and the binding portion of the aPLXNA1 mAb Fab, due to lack of well resolved density on the tail region of PLXNA1, and to highlight the only relevant information for the Fab binding PLXNA1 loci. Validation of the model was done through MolProbity (96) validation tool the built-in real-space refinement module of Phenix. Key interaction residues were generated using PDBePISA (97) and PyMOL (Schrödinger, LLC, 2015, the PyMOL Molecular Graphics System, https://www.pymol.org/). Model images were generated in ChimeraX and PyMOL.

### Quantification and statistical analysis

The statistical analysis for comparing two groups was performed using unpaired *t* tests (GraphPad Prism 9.0, https://www.graphpad.com/). Means were taken to be significantly different if *p* < 0.05. In figures, ∗ indicates *p* < 0.05, ∗∗ indicates *p* < 0.01, ∗∗∗ indicates *p* < 0.001, ∗∗∗∗ indicates *p* < 0.0001, and not significant (ns) indicates *p* ≥ 0.05 for the indicated pairwise comparison. Error bars in all figures indicate SEM unless stated otherwise.

## Data availability

All data in this study are included in this article. They are available from the corresponding author upon reasonable request.

The following atomic model and cryo-EM map reported in this work have been deposited in the PDB (https://www.rcsb.org/) and Electron Microscopy Data Bank (https://www.ebi.ac.uk/emdb/). Cryo-EM structure of aPLXNA1-19-43 Fab in complex with PLXNA1 dimer: 5L59; EMD-61131.

## Supporting information

This article contains supporting information.

## Conflict of interest

L. A. N., H. T., C. P. F., Z. X., L. B., Y. E. C., Y. G., L. Y., M. W. C., and Z. W. were employed or associated with aTyr Pharma when conducting research for this study. Pursuant to their employment, all current employees of aTyr Pharma have stock options in the company. L. A. N. owns stock in aTyr Pharma. The other authors declare that they have no conflicts of interest with the contents of this article.
