# Supplementary material for: A bispecific antibody designed to act as a NRP2/PLXNA1 agonist mimics anticancer activity of SEMA3F
Source: J Biol Chem. 2025 Dec 13;302(2):111056. doi: 10.1016/j.jbc.2025.111056 (PMC12809092; doi:10.1016/j.jbc.2025.111056)
Supplement: Supplementary Material 1 [file mmc1.docx]

**A Bispecific Antibody Designed to Act as a NRP2/PLXNA1 Agonist**

**Mimics Anti-cancer Activity of SEMA3F**

Honglei Tian^1,2,3^, Chun Po Fung^1,2,3^, Luke Burman^4^, Yeeting E. Chong^4^, Changdong Liu^2^, Yanyan Geng^1,2,3^, Lam Yang^1,2,3^, Man Wai Chow^1,2,3^, Yingyi Zhang^5^, Kwok Wa Hugo Ho^5^, Guang Zhu^2^, Zhenguo Wu^2^, Xiang-Lei Yang^6^, Zhiwen Xu^4^, and Leslie A. Nangle^4,*^

1. IAS HKUST-Scripps R&D Laboratory, Institute for Advanced Study, Hong Kong University of Science and Technology, Clear Water Bay, Kowloon, Hong Kong, China.

2. Division of Life Science, The Hong Kong University of Science and Technology, Hong Kong, China.

3. Pangu Biopharma, Hong Kong, China.

4. aTyr Pharma, San Diego, CA 92121, U.S.A.

5. Biological Cryo-EM Center, The Hong Kong University of Science and Technology, Hong Kong, China.

6. Department of Integrative Structural and Computational Biology, The Scripps Research Institute, La Jolla, CA 92037, U.S.A.

*Corresponding author, lnangle@atyrpharma.com

# **SUPPLEMENTARY FIGURES**


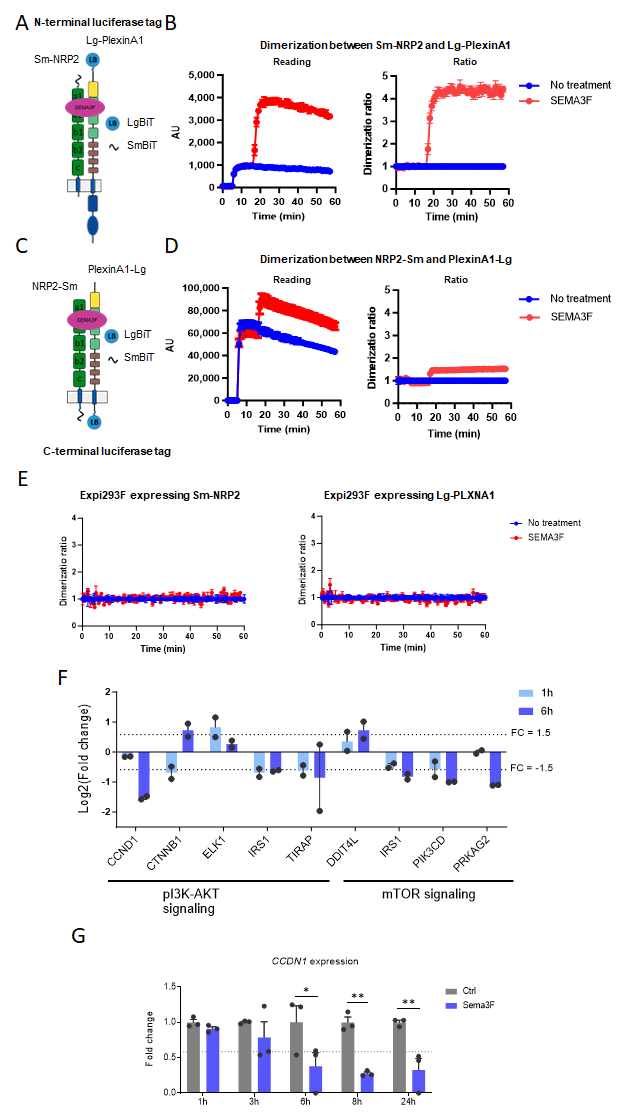


**Supplementary figure 1. SEMA3F induces dimerization of PLXNA1 and NRP2 and regulates gene expression**

A. Schematic illustrating split luciferase tags fused to the N-termini of PLXNA1 and NRP2.

B. Expi293F cells expressing Lg-PLXNA1 and Sm-NRP2 were treated with SEMA3F. Luminescence was measured, and the dimerization ratio was calculated.

C. Schematic illustrating split luciferase tags fused to the C-termini of PLXNA1 and NRP2.

D. Expi293F cells expressing PLXNA1-Lg and NRP2-Sm were treated with SEMA3F. Luminescence was measured, and the dimerization ratio was calculated.

E. Negative controls for the dimerization assay. Expi293F cells expressing Sm-NRP2 or Lg-PLXNA1 alone were treated with SEMA3F. Dimerization ratios were shown.

F. Gene expression analysis following SEMA3F treatment. The panel shows genes with a fold change greater than 1.5, as determined by qPCR assay.

G. Downregulation of CCND1 expression after 6 hours of SEMA3F treatment, as measured by qPCR.


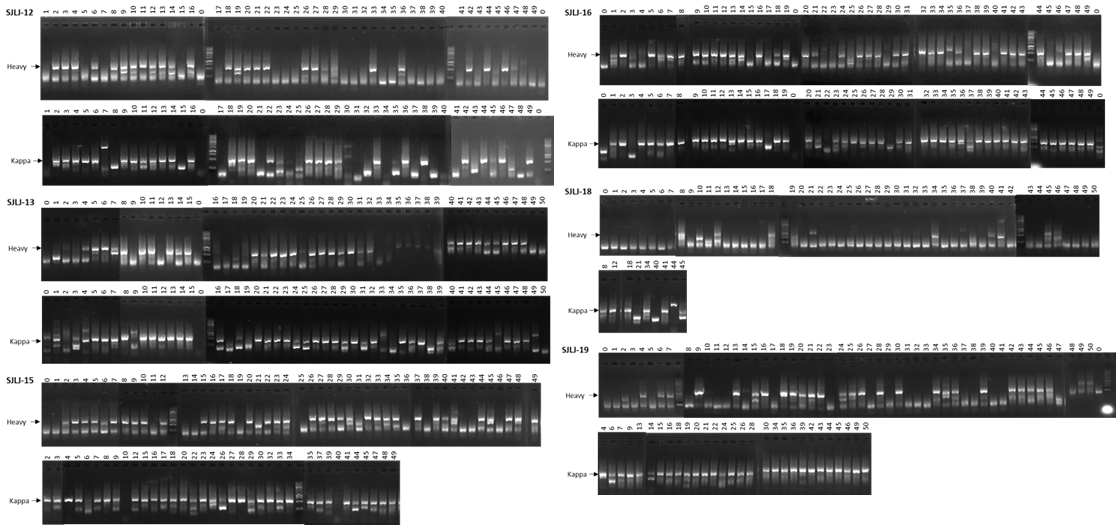


**Supplementary figure 2. Cloning of anti-PLXNA1 antibody heavy and light (kappa) variable regions from six mice (SJLJ-12, -13, -15, -16, -18, and -19).**

Arrows beside the agarose gels indicate the expected size of the correctly amplified genes.


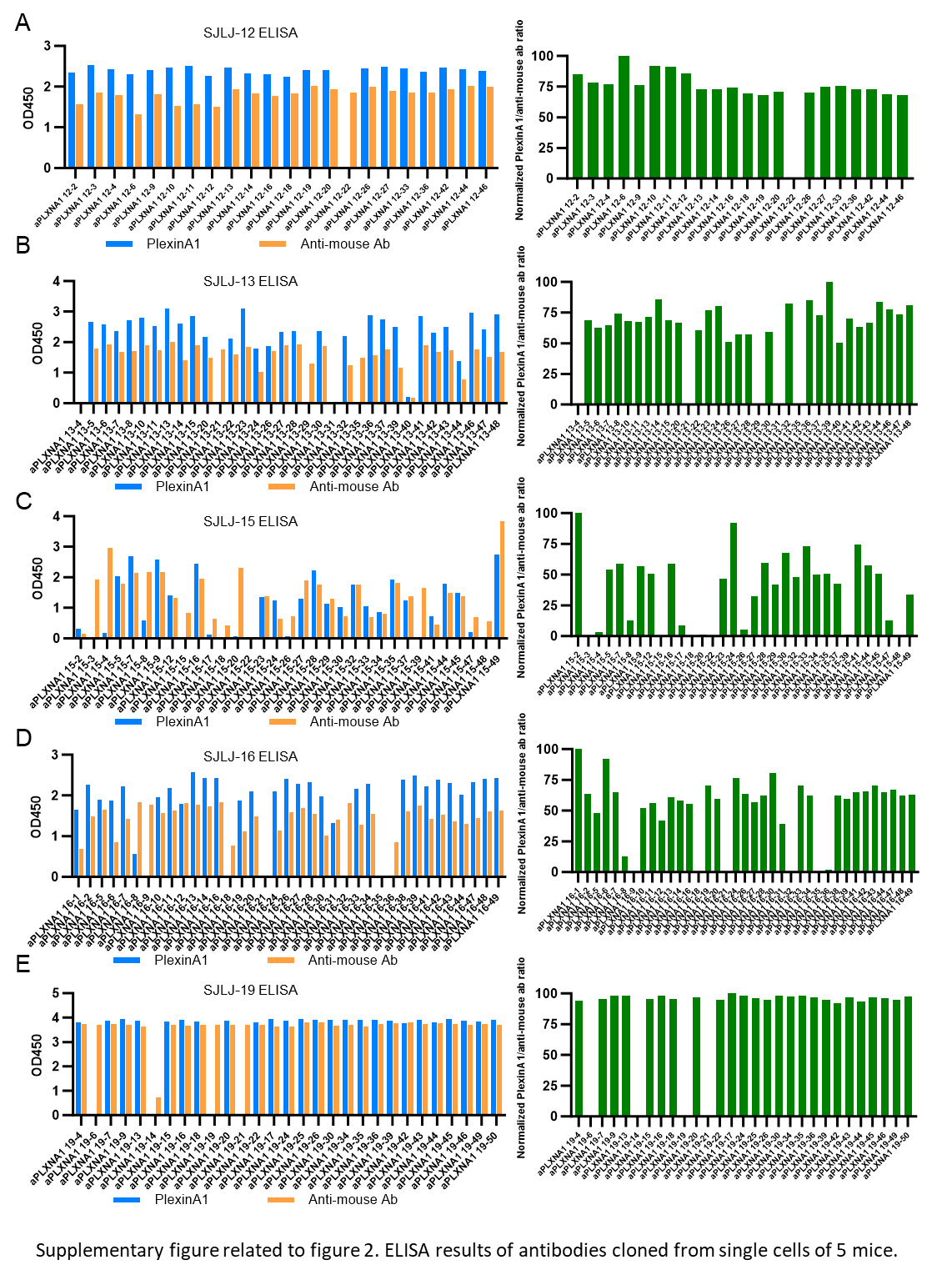


**Supplementary figure 3. Verification of anti-PLXNA1 antibodies by ELISA.**

Antibodies were expressed in Expi293F cells, and secreted antibodies in the culture medium were tested for binding to PLXNA1 and anti-mouse antibodies using ELISA.

A. ELISA results for antibodies cloned from the mouse SJLJ-12.

B. ELISA results for antibodies from the mouse SJLJ-13.

C. ELISA results for antibodies from the mouse SJLJ-15.

D. ELISA results for antibodies from the mouse SJLJ-16.

E. ELISA results for antibodies from the mouse SJLJ-19.


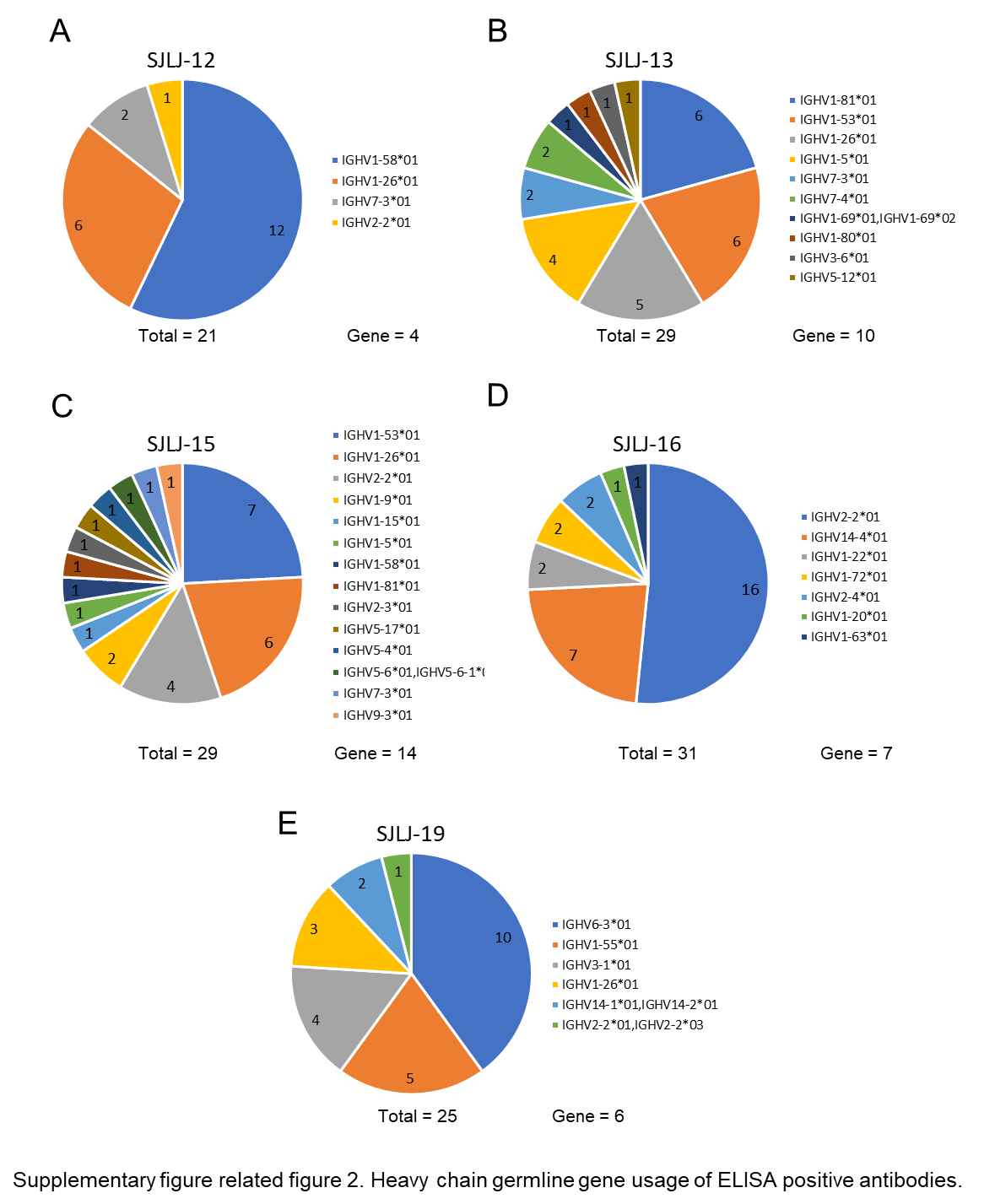


**Supplementary figure 4. Analysis of germline IGHV gene usage of anti-PLXNA1 antibody heavy chains**

A. Heavy chains from the mouse SJLJ-12.

B. Heavy chains from the mouse SJLJ-12.

C. Heavy chains from the mouse SJLJ-15.

D. Heavy chains from the mouse SJLJ-16.

E. Heavy chains from the mouse SJLJ-19.


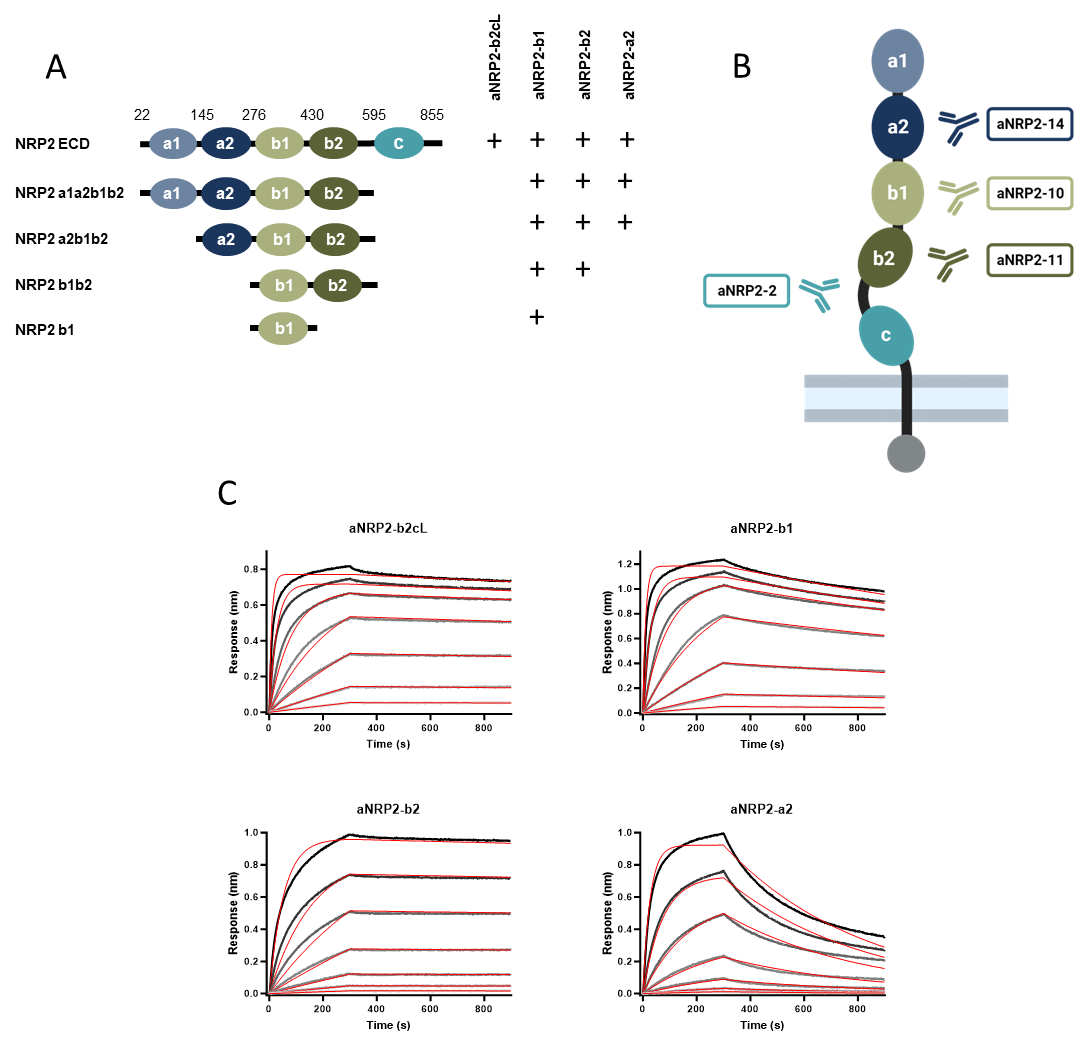


**Supplementary figure 5. Binding sites and affinities of aNRP2 antibodies.**

A. Mapping the binding region of anti-NRP2 antibodies using an ELISA assay.

B. Schematic representation of binding sites of anti-NRP2 antibodies.

C. Measuring affinity of anti-NRP2 antibodies to NRP2 protein. The Fab was immobilized using biosensor tips coated with anti-mouse kappa antibody and subsequently exposed to a concentration series of NRP2 (black and gray lines). Data were fitted to a 1:1 binding model (red lines) to calculate binding constants.


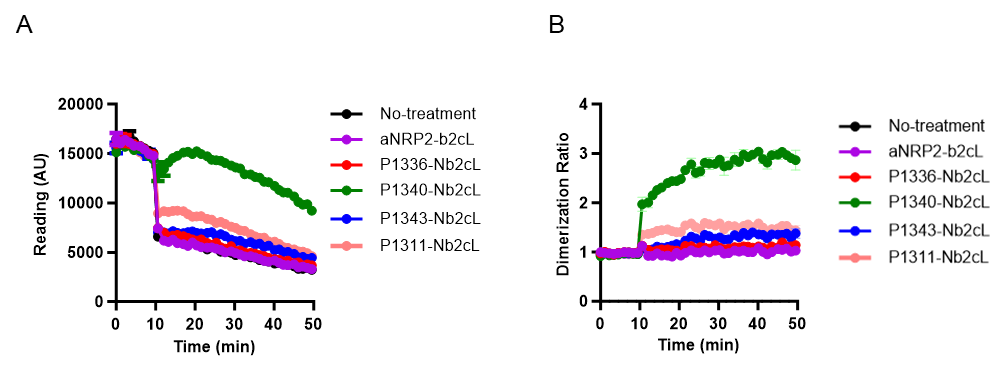


**Supplementary figure 6. Dimerization assay screening for bsAbs that dimerize PLXNA1 and NRP2.**

A. Expi293F cells expressing Lg-PLXNA1 and Sm-NRP2 were seeded in a white plate with luminescence substrate. Baseline luminescence was measured for 10 minutes. Subsequently, medium containing bsAbs was added to the wells, and luminescence was recorded for an additional 40 minutes.

B. The dimerization ratio from panel A was calculated to evaluate bsAb-induced dimerization.


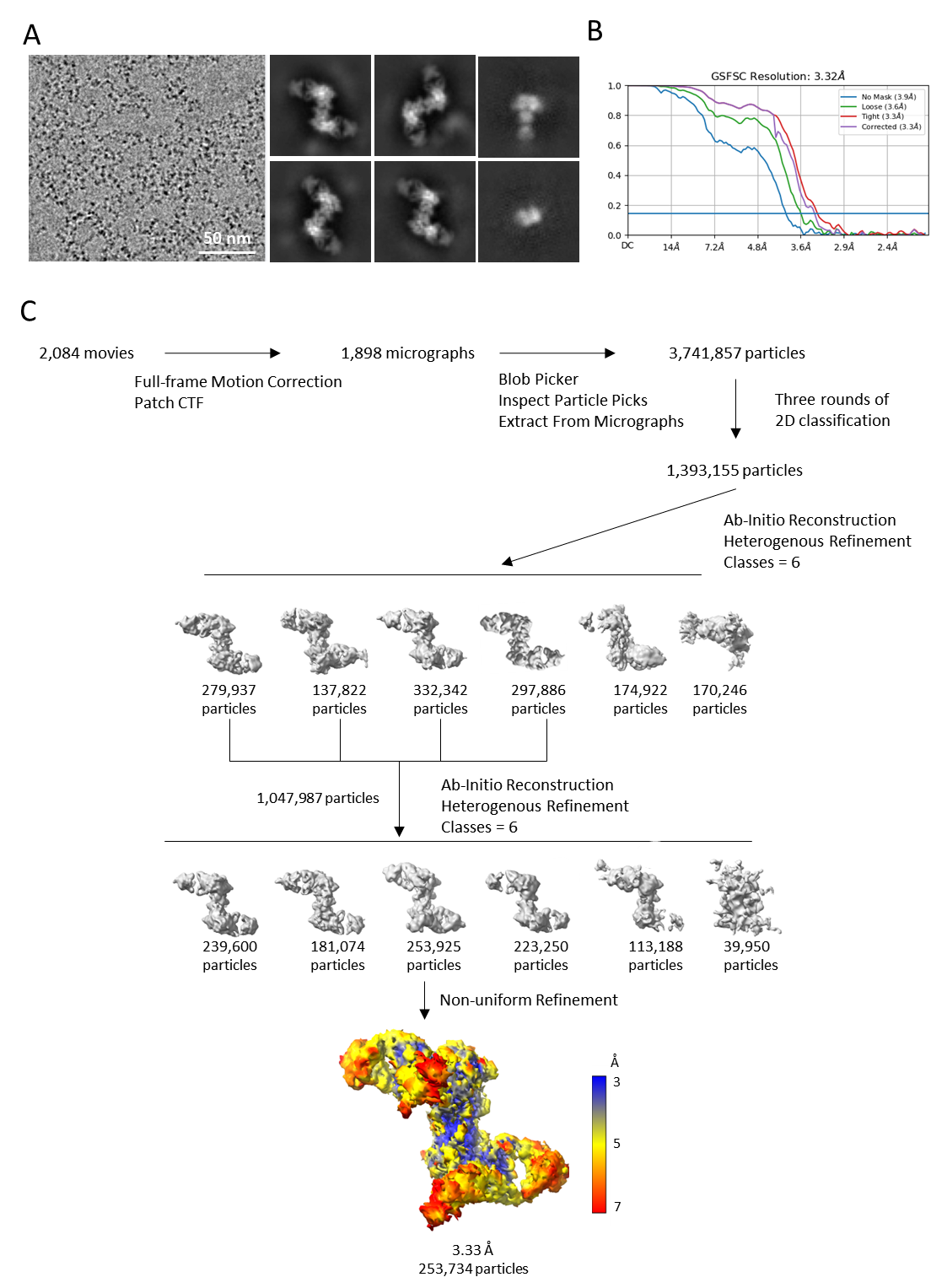


**Supplementary figure 7. Cryo-EM data processing of the PLXNA1-Fab complex.**

A. Representative cryo-EM micrograph and 2D class averages of the PLXNA1-Fab complex.

B. Resolution assessment of the PLXNA1-Fab complex density map.

C. Overview of the data processing workflow for the PLXNA1-Fab complex dataset.


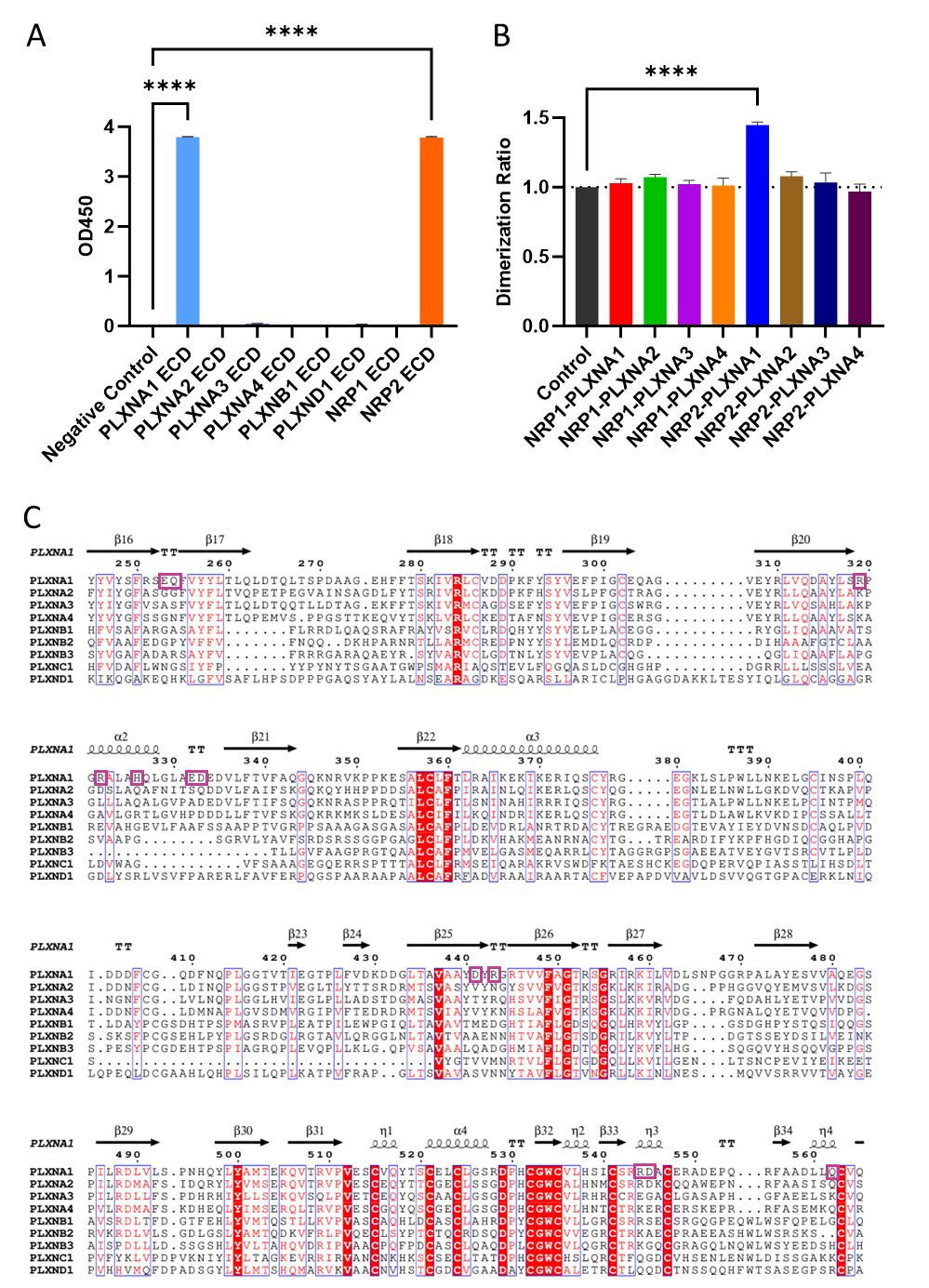


**Supplementary Figure 8. Specificity analysis of the bsAb P1943-Nb2cL.**

A. ELISA assessing binding of bsAb P1943-Nb2cL to the extracellular domains (ECDs) of PLXNA1-4, PLXNB1, PLXND1, NRP1, and NRP2. No protein coating was employed as the negative control. N = 3, **** p < 0.0001 by Student’s t test.

B. Dimerization ratios of NRP-PLXNA protein receptor pairs overexpressed on Expi293F cells upon treatment with the bsAb P1943-Nb2cL at 100 nM. Control is Expi293F cell. N = 3, **** p < 0.0001 by one-way ANOVA.

C. Sequence alignment of human plexin (PLXN) proteins from PLXNA1 aa245-495.

Pink boxes highlight the PLXNA1 critical residues for the bsAb P1943-Nb2cL binding. Blue framed and red residues: physico-chemical similarity >70% among PLXN proteins. White residue with red highlight: identical residue among PLXN proteins. Label above the sequence alignment indicates PLXNA1 secondary structure (PDB: 5l59). ɑ: alpha-helix; β: beta-sheet; η: 310-helix; T: turns.


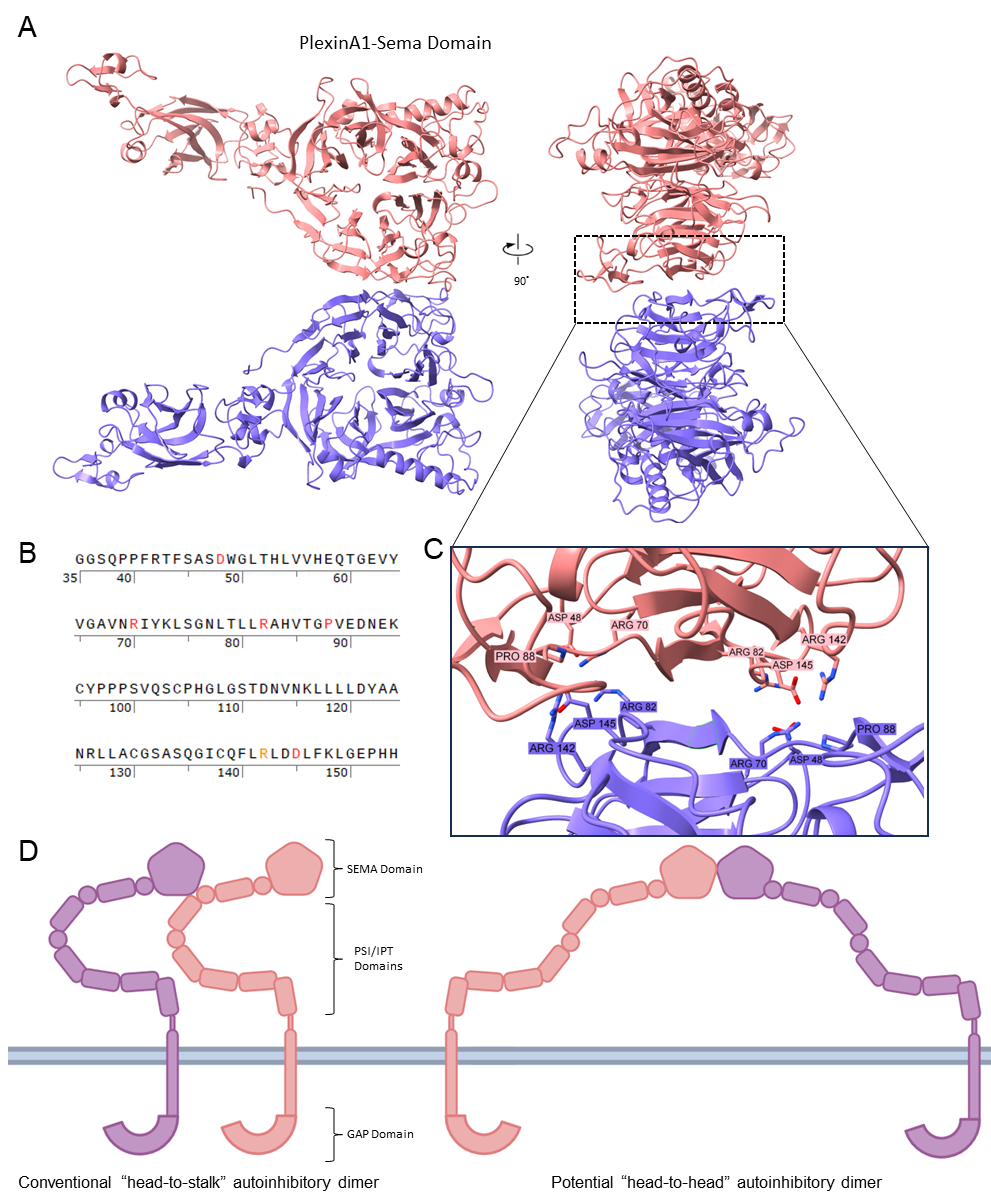


**Supplementary figure 9. Dimer interface of between two PLXNA1 sema domains.**

A. Overview of the PLXNA1 dimer, with one chain colored pink and the other purple.

B. Sequence of the PLXNA1 sema domain dimerization surface, with residues critical for interaction highlighted: red for potential salt bridges and orange for hydrogen bonds.

C. Detailed view of the interactions between PLXNA1 residues at the dimerization interface.

D. Comparison of the inhibitory dimer to the conventional PLXNA1 “head-to-stalk” inhibitory dimer.

**Supplementary Figure 10. Protein expression levels of NRP2 and PLXNA1 in renal cell carcinoma versus control.**

Data were obtained from the Human Protein Atlas. nRPX: normalized relative protein expression. Statistical analysis was performed using one-way ANOVA: **** p < 0.0001. NRP2 data source: https://www.proteinatlas.org/ENSG00000118257-NRP2/cancer. Sample number: N = 76 for normal samples and 101 for tumor samples; PLXNA1 data source: https://www.proteinatlas.org/ENSG00000114554-PLXNA1/cancer. Sample number: N = 84 for normal samples and 110 for tumor samples.

# **SUPPLEMENTARY TABLES**

**Supplementary table 1. Cancer cell lines information**

| **Cell line** | **Disease** | **Origin** |
| --- | --- | --- |
| HSC-2 | Oral cavity squamous cell carcinoma | Human 69Y Male |
| SAS | Tongue squamous cell carcinoma | Human 69Y Female |
| H157 | Buccal mucosa squamous cell carcinoma | Human 84Y Male |
| 786-O | Renal cell carcinoma | Human 58Y Male |
| Caki-1 | Clear cell renal cell carcinoma | Human 49Y Male |
| U-251MG | Glioblastoma | Human 75Y Male |

**Supplementary table 2. PLXNA1 and NRP2 expression in cancer cell lines in this study**

| **Gene** | **Cell Line** | **TPM** | **pTPM** | **nTPM** |
| --- | --- | --- | --- | --- |
| NRP2 | U-251MG | 247.4 | 310.3 | 289.1 |
| NRP2 | Caki-1 | 76.2 | 95.2 | 99.1 |
| NRP2 | HSC-2 | 6.6 | 8 | 16 |
| NRP2 | 786-O | 110.4 | 135.3 | 132.8 |
| NRP2 | NCI-H1573 | 0.5 | 0.7 | 0.6 |
| PLXNA1 | U-251MG | 22.1 | 27.6 | 26 |
| PLXNA1 | Caki-1 | 23.7 | 29.4 | 32.2 |
| PLXNA1 | HSC-2 | 35.1 | 42.5 | 85.3 |
| PLXNA1 | 786-O | 16.8 | 20.6 | 20.2 |
| PLXNA1 | NCI-H1573 | 7.9 | 10 | 8.6 |

**Supplementary table 3. qPCR screening for SEMA3F regulated genes in PI3K-AKT signalling pathway.**

| **No.** | **Position** | **UniGene** | **GenBank** | **Symbol** | **Fold change at 1 h** | **Fold change at 6 h** |
| --- | --- | --- | --- | --- | --- | --- |
| 1 | A01 | Hs.12341 | NM_001111 | ADAR | -0.241 | 0.097 |
| 2 | A02 | Hs.525622 | NM_005163 | AKT1 | 0.043 | 0.040 |
| 3 | A03 | Hs.631535 | NM_001626 | AKT2 | -0.063 | 0.078 |
| 4 | A04 | Hs.498292 | NM_005465 | AKT3 | -0.051 | 0.312 |
| 5 | A05 | Hs.158932 | NM_000038 | APC | -0.275 | -0.194 |
| 6 | A06 | Hs.370254 | NM_004322 | BAD | -0.039 | 0.012 |
| 7 | A07 | Hs.159494 | NM_000061 | BTK | ND | ND |
| 8 | A08 | Hs.329502 | NM_001229 | CASP9 | -0.309 | -0.025 |
| 9 | A09 | Hs.523852 | NM_053056 | CCND1 | -0.152 | -1.526 |
| 10 | A10 | Hs.163867 | NM_000591 | CD14 | 0.233 | ND |
| 11 | A11 | Hs.690198 | NM_001791 | CDC42 | -0.019 | -0.092 |
| 12 | A12 | Hs.238990 | NM_004064 | CDKN1B | -0.195 | 0.382 |
| 13 | B01 | Hs.198998 | NM_001278 | CHUK | -0.025 | -0.088 |
| 14 | B02 | Hs.644056 | NM_001895 | CSNK2A1 | 0.089 | -0.016 |
| 15 | B03 | Hs.476018 | NM_001904 | CTNNB1 | -0.691 | 0.731 |
| 16 | B04 | Hs.131431 | NM_002759 | EIF2AK2 | 0.221 | 0.205 |
| 17 | B05 | Hs.648394 | NM_001417 | EIF4B | -0.088 | 0.150 |
| 18 | B06 | Hs.249718 | NM_001968 | EIF4E | ND | ND |
| 19 | B07 | Hs.411641 | NM_004095 | EIF4EBP1 | -0.050 | -0.009 |
| 20 | B08 | Hs.433750 | NM_182917 | EIF4G1 | -0.318 | -0.133 |
| 21 | B09 | Hs.181128 | NM_005229 | ELK1 | 0.826 | 0.267 |
| 22 | B10 | Hs.2007 | NM_000639 | FASLG | ND | ND |
| 23 | B11 | Hs.471933 | NM_000801 | FKBP1A | -0.309 | 0.246 |
| 24 | B12 | Hs.728789 | NM_005252 | FOS | 0.023 | -0.118 |
| 25 | C01 | Hs.370666 | NM_002015 | FOXO1 | -0.378 | 0.146 |
| 26 | C02 | Hs.220950 | NM_001455 | FOXO3 | 0.039 | 0.408 |
| 27 | C03 | Hs.74471 | NM_000165 | GJA1 | -0.151 | -0.054 |
| 28 | C04 | Hs.164060 | NM_005311 | GRB10 | -0.072 | 0.282 |
| 29 | C05 | Hs.444356 | NM_002086 | GRB2 | -0.359 | 0.075 |
| 30 | C06 | Hs.445733 | NM_002093 | GSK3B | -0.118 | 0.139 |
| 31 | C07 | Hs.37003 | NM_005343 | HRAS | -0.031 | -0.141 |
| 32 | C08 | Hs.520973 | NM_001540 | HSPB1 | 0.548 | 0.135 |
| 33 | C09 | Hs.160562 | NM_000618 | IGF1 | 0.497 | ND |
| 34 | C10 | Hs.643120 | NM_000875 | IGF1R | -0.053 | 0.294 |
| 35 | C11 | Hs.5158 | NM_004517 | ILK | -0.085 | 0.012 |
| 36 | C12 | Hs.522819 | NM_001569 | IRAK1 | -0.395 | -0.147 |
| 37 | D01 | Hs.471508 | NM_005544 | IRS1 | -0.691 | -0.628 |
| 38 | D02 | Hs.643813 | NM_002211 | ITGB1 | 0.059 | 0.025 |
| 39 | D03 | Hs.714791 | NM_002228 | JUN | -0.523 | -0.479 |
| 40 | D04 | Hs.145442 | NM_002755 | MAP2K1 | -0.268 | 0.174 |
| 41 | D05 | Hs.431850 | NM_002745 | MAPK1 | -0.090 | 0.045 |
| 42 | D06 | Hs.485233 | NM_001315 | MAPK14 | 0.209 | 0.215 |
| 43 | D07 | Hs.861 | NM_002746 | MAPK3 | -0.193 | 0.136 |
| 44 | D08 | Hs.138211 | NM_002750 | MAPK8 | -0.020 | 0.210 |
| 45 | D09 | Hs.6917 | NM_001018025 | MTCP1 | -0.231 | -0.236 |
| 46 | D10 | Hs.338207 | NM_004958 | MTOR | -0.096 | 0.018 |
| 47 | D11 | Hs.82116 | NM_002468 | MYD88 | 0.069 | 0.305 |
| 48 | D12 | Hs.654408 | NM_003998 | NFKB1 | -0.167 | 0.105 |
| 49 | E01 | Hs.81328 | NM_020529 | NFKBIA | -0.052 | -0.081 |
| 50 | E02 | Hs.387804 | NM_002568 | PABPC1 | 0.096 | 0.126 |
| 51 | E03 | Hs.435714 | NM_002576 | PAK1 | -0.173 | 0.178 |
| 52 | E04 | Hs.74615 | NM_006206 | PDGFRA | 0.103 | 0.143 |
| 53 | E05 | Hs.470633 | NM_002610 | PDK1 | 0.003 | -0.059 |
| 54 | E06 | Hs.256667 | NM_002611 | PDK2 | -0.083 | 0.476 |
| 55 | E07 | Hs.459691 | NM_002613 | PDPK1 | 0.007 | -0.036 |
| 56 | E08 | Hs.553498 | NM_006218 | PIK3CA | -0.022 | 0.082 |
| 57 | E09 | Hs.32942 | NM_002649 | PIK3CG | ND | ND |
| 58 | E10 | Hs.132225 | NM_181504 | PIK3R1 | -0.194 | 0.392 |
| 59 | E11 | Hs.371344 | NM_005027 | PIK3R2 | -0.062 | 0.559 |
| 60 | E12 | Hs.531704 | NM_002737 | PRKCA | -0.104 | 0.334 |
| 61 | F01 | Hs.460355 | NM_002738 | PRKCB | ND | ND |
| 62 | F02 | Hs.496255 | NM_002744 | PRKCZ | -0.132 | 0.080 |
| 63 | F03 | Hs.500466 | NM_000314 | PTEN | 0.057 | 0.302 |
| 64 | F04 | Hs.395482 | NM_005607 | PTK2 | -0.184 | 0.186 |
| 65 | F05 | Hs.506852 | NM_002834 | PTPN11 | -0.138 | 0.157 |
| 66 | F06 | Hs.413812 | NM_006908 | RAC1 | -0.074 | -0.034 |
| 67 | F07 | Hs.159130 | NM_002880 | RAF1 | -0.115 | -0.062 |
| 68 | F08 | Hs.664080 | NM_002890 | RASA1 | -0.189 | 0.089 |
| 69 | F09 | Hs.513609 | NM_005611 | RBL2 | -0.058 | 0.263 |
| 70 | F10 | Hs.283521 | NM_005614 | RHEB | 0.019 | -0.048 |
| 71 | F11 | Hs.247077 | NM_001664 | RHOA | 0.062 | 0.053 |
| 72 | F12 | Hs.149957 | NM_002953 | RPS6KA1 | -0.196 | 0.045 |
| 73 | G01 | Hs.463642 | NM_003161 | RPS6KB1 | -0.146 | 0.051 |
| 74 | G02 | Hs.433795 | NM_003029 | SHC1 | -0.089 | -0.036 |
| 75 | G03 | Hs.592839 | NM_005633 | SOS1 | -0.114 | 0.098 |
| 76 | G04 | Hs.520140 | NM_003131 | SRF | -0.193 | -0.471 |
| 77 | G05 | Hs.2484 | NM_021966 | TCL1A | ND | ND |
| 78 | G06 | Hs.537126 | NM_001039661 | TIRAP | -0.613 | ND |
| 79 | G07 | Hs.174312 | NM_138554 | TLR4 | 0.028 | 0.021 |
| 80 | G08 | Hs.368527 | NM_019009 | TOLLIP | -0.066 | 0.182 |
| 81 | G09 | Hs.370854 | NM_000368 | TSC1 | 0.008 | 0.186 |
| 82 | G10 | Hs.90303 | NM_000548 | TSC2 | -0.371 | 0.235 |
| 83 | G11 | Hs.143728 | NM_003941 | WASL | -0.519 | 0.303 |
| 84 | G12 | Hs.226755 | NM_003405 | YWHAH | 0.079 | -0.092 |

**Supplementary table 4. qPCR screening for SEMA3F regulated genes in mTOR signalling pathway**

| **No.** | **Position** | **UniGene** | **GenBank** | **Symbol** | **Fold change at 1 h** | **Fold change at 6 h** |
| --- | --- | --- | --- | --- | --- | --- |
| 1 | A01 | Hs.525622 | NM_005163 | AKT1 | -0.029 | -0.251 |
| 2 | A02 | Hs.515542 | NM_032375 | AKT1S1 | -0.035 | -0.371 |
| 3 | A03 | Hs.631535 | NM_001626 | AKT2 | -0.071 | 0.309 |
| 4 | A04 | Hs.498292 | NM_005465 | AKT3 | -0.096 | 0.280 |
| 5 | A05 | Hs.632536 | NM_016289 | CAB39 | -0.097 | -0.080 |
| 6 | A06 | Hs.87159 | NM_030925 | CAB39L | 0.169 | 0.184 |
| 7 | A07 | Hs.690198 | NM_001791 | CDC42 | -0.090 | -0.023 |
| 8 | A08 | Hs.198998 | NM_001278 | CHUK | 0.161 | -0.161 |
| 9 | A09 | Hs.523012 | NM_019058 | DDIT4 | -0.116 | 0.062 |
| 10 | A10 | Hs.480378 | NM_145244 | DDIT4L | 0.357 | 0.731 |
| 11 | A11 | Hs.112981 | NM_022783 | DEPTOR | 0.162 | 0.386 |
| 12 | A12 | Hs.648394 | NM_001417 | EIF4B | -0.030 | 0.193 |
| 13 | B01 | Hs.249718 | NM_001968 | EIF4E | ND | ND |
| 14 | B02 | Hs.411641 | NM_004095 | EIF4EBP1 | 0.098 | -0.231 |
| 15 | B03 | Hs.730236 | NM_004096 | EIF4EBP2 | 0.017 | 0.302 |
| 16 | B04 | Hs.471933 | NM_000801 | FKBP1A | 0.005 | -0.341 |
| 17 | B05 | Hs.173464 | NM_012181 | FKBP8 | -0.067 | 0.105 |
| 18 | B06 | Hs.445733 | NM_002093 | GSK3B | 0.042 | 0.191 |
| 19 | B07 | Hs.597216 | NM_001530 | HIF1A | 0.125 | 0.409 |
| 20 | B08 | Hs.37003 | NM_005343 | HRAS | 0.084 | -0.079 |
| 21 | B09 | Hs.90093 | NM_002154 | HSPA4 | 0.022 | -0.176 |
| 22 | B10 | Hs.160562 | NM_000618 | IGF1 | ND | ND |
| 23 | B11 | Hs.450230 | NM_000598 | IGFBP3 | 0.059 | 0.122 |
| 24 | B12 | Hs.597664 | NM_001556 | IKBKB | 0.107 | 0.173 |
| 25 | C01 | Hs.5158 | NM_004517 | ILK | 0.030 | -0.144 |
| 26 | C02 | Hs.654579 | NM_000207 | INS | ND | ND |
| 27 | C03 | Hs.465744 | NM_000208 | INSR | -0.226 | 0.148 |
| 28 | C04 | Hs.471508 | NM_005544 | IRS1 | -0.453 | -0.823 |
| 29 | C05 | Hs.431850 | NM_002745 | MAPK1 | -0.012 | -0.183 |
| 30 | C06 | Hs.861 | NM_002746 | MAPK3 | 0.045 | 0.260 |
| 31 | C07 | Hs.495138 | NM_024117 | MAPKAP1 | 0.100 | 0.017 |
| 32 | C08 | Hs.29203 | NM_022372 | MLST8 | 0.183 | 0.142 |
| 33 | C09 | Hs.338207 | NM_004958 | MTOR | 0.146 | 0.280 |
| 34 | C10 | Hs.286226 | NM_033375 | MYO1C | 0.189 | -0.113 |
| 35 | C11 | Hs.459691 | NM_002613 | PDPK1 | 0.079 | 0.186 |
| 36 | C12 | Hs.464971 | NM_002647 | PIK3C3 | 0.061 | 0.176 |
| 37 | D01 | Hs.553498 | NM_006218 | PIK3CA | -0.040 | -0.078 |
| 38 | D02 | Hs.239818 | NM_006219 | PIK3CB | 0.038 | 0.166 |
| 39 | D03 | Hs.518451 | NM_005026 | PIK3CD | -0.574 | -1.000 |
| 40 | D04 | Hs.32942 | NM_002649 | PIK3CG | ND | ND |
| 41 | D05 | Hs.382865 | NM_002662 | PLD1 | 0.125 | 0.354 |
| 42 | D06 | Hs.104519 | NM_002663 | PLD2 | -0.052 | 0.377 |
| 43 | D07 | Hs.483408 | NM_002715 | PPP2CA | 0.005 | -0.007 |
| 44 | D08 | Hs.655213 | NM_181678 | PPP2R2B | 0.015 | -0.324 |
| 45 | D09 | Hs.400740 | NM_021131 | PPP2R4 | 0.051 | 0.177 |
| 46 | D10 | Hs.43322 | NM_006251 | PRKAA1 | 0.125 | 0.150 |
| 47 | D11 | Hs.437039 | NM_006252 | PRKAA2 | -0.176 | 0.131 |
| 48 | D12 | Hs.715515 | NM_006253 | PRKAB1 | 0.255 | 0.235 |
| 49 | E01 | Hs.50732 | NM_005399 | PRKAB2 | -0.084 | -0.166 |
| 50 | E02 | Hs.530862 | NM_002733 | PRKAG1 | -0.063 | -0.044 |
| 51 | E03 | Hs.647072 | NM_016203 | PRKAG2 | 0.002 | -1.105 |
| 52 | E04 | Hs.591634 | NM_017431 | PRKAG3 | ND | ND |
| 53 | E05 | Hs.531704 | NM_002737 | PRKCA | 0.074 | 0.366 |
| 54 | E06 | Hs.460355 | NM_002738 | PRKCB | ND | ND |
| 55 | E07 | Hs.580351 | NM_005400 | PRKCE | -0.015 | 0.319 |
| 56 | E08 | Hs.631564 | NM_002739 | PRKCG | ND | 0.487 |
| 57 | E09 | Hs.500466 | NM_000314 | PTEN | 0.169 | -0.199 |
| 58 | E10 | Hs.283521 | NM_005614 | RHEB | 0.128 | 0.144 |
| 59 | E11 | Hs.247077 | NM_001664 | RHOA | 0.226 | -0.043 |
| 60 | E12 | Hs.407926 | NM_152756 | RICTOR | -0.051 | 0.205 |
| 61 | F01 | Hs.408073 | NM_001010 | RPS6 | 0.030 | -0.032 |
| 62 | F02 | Hs.149957 | NM_002953 | RPS6KA1 | 0.084 | -0.152 |
| 63 | F03 | Hs.655277 | NM_021135 | RPS6KA2 | 0.113 | 0.399 |
| 64 | F04 | Hs.510225 | NM_004755 | RPS6KA5 | -0.170 | 0.303 |
| 65 | F05 | Hs.463642 | NM_003161 | RPS6KB1 | 0.048 | 0.144 |
| 66 | F06 | Hs.534345 | NM_003952 | RPS6KB2 | -0.091 | 0.250 |
| 67 | F07 | Hs.133044 | NM_020761 | RPTOR | ND | 0.066 |
| 68 | F08 | Hs.432330 | NM_006570 | RRAGA | 0.023 | -0.130 |
| 69 | F09 | Hs.50282 | NM_006064 | RRAGB | 0.153 | 0.407 |
| 70 | F10 | Hs.532461 | NM_022157 | RRAGC | 0.281 | 0.225 |
| 71 | F11 | Hs.31712 | NM_021244 | RRAGD | 0.021 | 0.122 |
| 72 | F12 | Hs.510078 | NM_005627 | SGK1 | 0.290 | -0.141 |
| 73 | G01 | Hs.515005 | NM_000455 | STK11 | -0.295 | 0.123 |
| 74 | G02 | Hs.652338 | NM_018571 | STRADB | -0.056 | -0.093 |
| 75 | G03 | Hs.271044 | NM_016111 | TELO2 | -0.537 | -0.534 |
| 76 | G04 | Hs.654481 | NM_000546 | TP53 | 0.082 | 0.103 |
| 77 | G05 | Hs.370854 | NM_000368 | TSC1 | 0.130 | 0.074 |
| 78 | G06 | Hs.90303 | NM_000548 | TSC2 | -0.178 | 0.106 |
| 79 | G07 | Hs.47061 | NM_003565 | ULK1 | 0.327 | -0.011 |
| 80 | G08 | Hs.168762 | NM_014683 | ULK2 | -0.345 | 0.302 |
| 81 | G09 | Hs.73793 | NM_003376 | VEGFA | 0.112 | 0.212 |
| 82 | G10 | Hs.78781 | NM_003377 | VEGFB | -0.203 | -0.142 |
| 83 | G11 | Hs.435215 | NM_005429 | VEGFC | -0.144 | 0.315 |
| 84 | G12 | Hs.74405 | NM_006826 | YWHAQ | 0.168 | 0.275 |

**Supplementary table 5. Summary of cell-based assays results**

| **No.** | **aNRP2 half** | **aPlexinA1 half** | **p-Akt level** | **CCND1 level** | **Viability%** |
| --- | --- | --- | --- | --- | --- |
| 1 | aNRP2-b2cL | aPlexinA1-12-10 | 1.22 | 1.21 | 101 |
| 2 |  | aPlexinA1-13-13 | 1.11 | 5.74 | 59 |
| 3 |  | aPlexinA1-13-26 | 0.69 | 3.46 | 68 |
| 4 |  | aPlexinA1-13-27 | 2.06 | 2.49 | 134 |
| 5 |  | aPlexinA1-13-40 | 1.68 | 1.64 | 91 |
| 6 |  | aPlexinA1-15-17 | 1.12 | 2.30 | 101 |
| 7 |  | aPlexinA1-15-02 | 1.40 | 1.75 | 95 |
| 8 |  | aPlexinA1-15-30 | 1.33 | 1.92 | 95 |
| 9 |  | aPlexinA1-15-47 | 1.11 | 2.06 | 110 |
| 10 |  | aPlexinA1-16-12 | 1.16 | 2.46 | 117 |
| 11 |  | aPlexinA1-16-14 | 0.79 | 2.26 | 72 |
| 12 |  | aPlexinA1-16-16 | 1.48 | 3.13 | 96 |
| 13 |  | aPlexinA1-16-39 | 1.51 | 3.86 | 107 |
| 14 |  | aPlexinA1-16-48 | 1.00 | 2.03 | 130 |
| 15 |  | aPlexinA1-19-15 | 0.94 | 13.27 | 99 |
| 16 |  | aPlexinA1-19-39 | 1.03 | 2.18 | 104 |
| 17 |  | aPlexinA1-19-04 | 1.81 | 1.39 | 107 |
| 18 |  | aPlexinA1-19-43 | 0.86 | 0.70 | 68 |
| 19 |  | aPlexinA1-19-07 | 1.30 | 1.41 | 127 |
| 20 | aNRP2-b1 | aPlexinA1-13-13 | 1.36 | 1.07 | 122 |
| 21 |  | aPlexinA1-13-26 | 1.48 | 1.32 | 117 |
| 22 |  | aPlexinA1-13-40 | 2.55 | 1.86 | 138 |
| 23 |  | aPlexinA1-15-30 | 2.10 | 2.96 | 114 |
| 24 | aNRP2-b2 | aPlexinA1-13-26 | 2.40 | 1.61 | 121 |
| 25 |  | aPlexinA1-13-40 | 2.01 | 0.15 | 83 |
| 26 |  | aPlexinA1-15-41 | 2.04 | 0.83 | 123 |
| 27 | aNRP2-a2 | aPlexinA1-12-06 | 2.95 | 25.28 | 118 |
| 28 |  | aPlexinA1-13-13 | 2.62 | 19.33 | 144 |
| 29 |  | aPlexinA1-13-26 | 2.53 | 20.36 | 112 |
| 30 |  | aPlexinA1-13-27 | 3.81 | 1.11 | 100 |
| 31 |  | aPlexinA1-13-40 | 2.30 | 1.06 | 71 |
| 32 |  | aPlexinA1-15-30 | 1.86 | 0.29 | 80 |
| 33 |  | aPlexinA1-15-47 | 2.29 | 11.58 | 104 |
| 34 |  | aPlexinA1-16-10 | 3.14 | 2.14 | 102 |
| 35 |  | aPlexinA1-16-12 | 1.86 | 0.96 | 112 |
| 36 |  | aPlexinA1-19-15 | 2.27 | 1.40 | 103 |
| 37 |  | aPlexinA1-19-43 | 1.23 | 0.67 | 100 |
| 38 |  | aPlexinA1-19-07 | 2.81 | 1.09 | 90 |
| 39 |  | No treatment | 1 | 1 | 100 |
| 40 |  | SEMA3F | 0.29 | 0.63 | 72 |

**Supplementary table 6. Cryo-EM data collection, processing, and model building**

| **Data collection** |  |
| --- | --- |
| EM equipment | Titan Krios |
| Voltage(kV) | 300 |
| Camera | Gatan K3 summit |
| Magnification | 81,000x |
| Electron dose (e/Å²) | 50 |
| Defocus range (μm) | -1.0~-2.5 |
| Pixel size (Å) | 1.026 |
| Collected movies | 2084 |
| **Reconstruction** |  |
| Software | CryoSPARC v4.1.2 |
| Final particles | 985,072 |
| B-factors (Å2 ) | 131.3 |
| Map resolution (Å) | 2.72(0.143) |
| **Atomic modelling** |  |
| Software | ChimeraX 1.3, Coot 0.9.5, Phenix 1.20 |
| Chain | 3 |
| Residues | 1095 |
| Water | 0 |
| Atoms | 8480 (Hydrogens:0) |
| RMSD Length (Å) | 0.002 |
| RMSD Angles (Å) | 0.557 |
| **Ramachandran plot (%)** |  |
| Favoured | 95 |
| Allowed | 5 |
| Outliers | 0 |
| Rotamer outliers | 4 |
| C-beta outliers | 0 |

**Supplementary table 7. Variable domain sequences of the bsAb P1943-Nb2cL**

| **bsAb half** | **Chain** | **Amino acid sequence of the variable domain** |
| --- | --- | --- |
| aPLXNA1 P1943 | Heavy chain | EVQLQQSGPELVKPGASVKISCKASGYKFTENYMDWVKQSHGESLEWIGDISPDNGDTSYNQKFRDKATLTVDKSSSTAYMELRSLTSEDSAVYYCAQIIYYDYVGYALDYWGQGTSVTVSS |
|  | Light chain kappa | QIVLTQSPAILSASPGEKVTMSCSVSSSITYMHWYQQKPGTSPKRWIYDTSKLASGVPARFSGSGSGTSFSLTISNMEAEDAATYYCHQRSSYPYSFGGGTKLEIK |
| aNRP2 Nb2cL | Heavy chain | QVRLQQPGAELVMPGASVKLSCKASGYTFTSYWMHWVKQRPGQGLEWIGVIHPNSASTFYNERFKTKATLTVDRSSSTAYMQLSSLTSEDSAVYYCSRPGTVRRSDYWGQGTTLTVSS |
|  | Light chain kappa | DVLMTQSPLSLSVSLGDQASISCRSSQNIVHSTGNTYLEWYLQKPGQSPKLLIYKVSNRFSGVPDRFSGSGSGTDFTLKISRVEAEDLGVYYCFQGSHVPWTFGGGTKLEIK |
